# Supplementary material for: Sex-specific lipidomic signatures in aortic valve disease reflect differential fibro-calcific progression
Source: Nat Commun. 2025 Jun 3;16:5163. doi: 10.1038/s41467-025-60411-2 (PMC12134101; doi:10.1038/s41467-025-60411-2)
Supplement: Supplementary file 1 — Supplementary Information [file 41467_2025_60411_MOESM1_ESM.pdf]

Sex-specific lipidomic signatures in aortic valve disease reflect differential fibro-calcific progression

Patricia Prabutzki[1#], Michele Wölk[2#], Julia Böttner[3], Zhixu Ni[2], Sarah Werner[3], Holger Thiele[3], Jürgen Schiller[1], Petra Büttner[3], Florian Schlotter[3,4*], Maria Fedorova[2*]

[1] Leipzig University, Faculty of Medicine, Institute for Medical Physics and Biophysics, Leipzig, Germany

[2] Center of Membrane Biochemistry and Lipid Research, University Hospital and Faculty of Medicine Carl Gustav Carus of TU Dresden, Dresden, Germany

[3] Heart Center Leipzig at Leipzig University, Department of Internal Medicine/Cardiology, Leipzig, Germany

[4] Department of Cardiology, University Medical Center of the Johannes Gutenberg University Mainz and German Center for Cardiovascular Research - Partner Site Rhine-Main, Mainz, Germany

[#]Authors contributed equally to this work

*Florian Schlotter, Department of Cardiology, University Medical Center of the Johannes Gutenberg University Mainz and German Center for Cardiovascular Research - Partner Site Rhine-Main, Mainz, Germany; E-mail: schlottf@uni-mainz.de

*Maria Fedorova, Lipid Metabolism: Analysis and Integration, Center of Membrane Biochemistry and Lipid Research, University Hospital Carl Gustav Carus and Faculty of Medicine of TU Dresden, Dresden, Germany; E-mail: maria.fedorova@tu-dresden.de

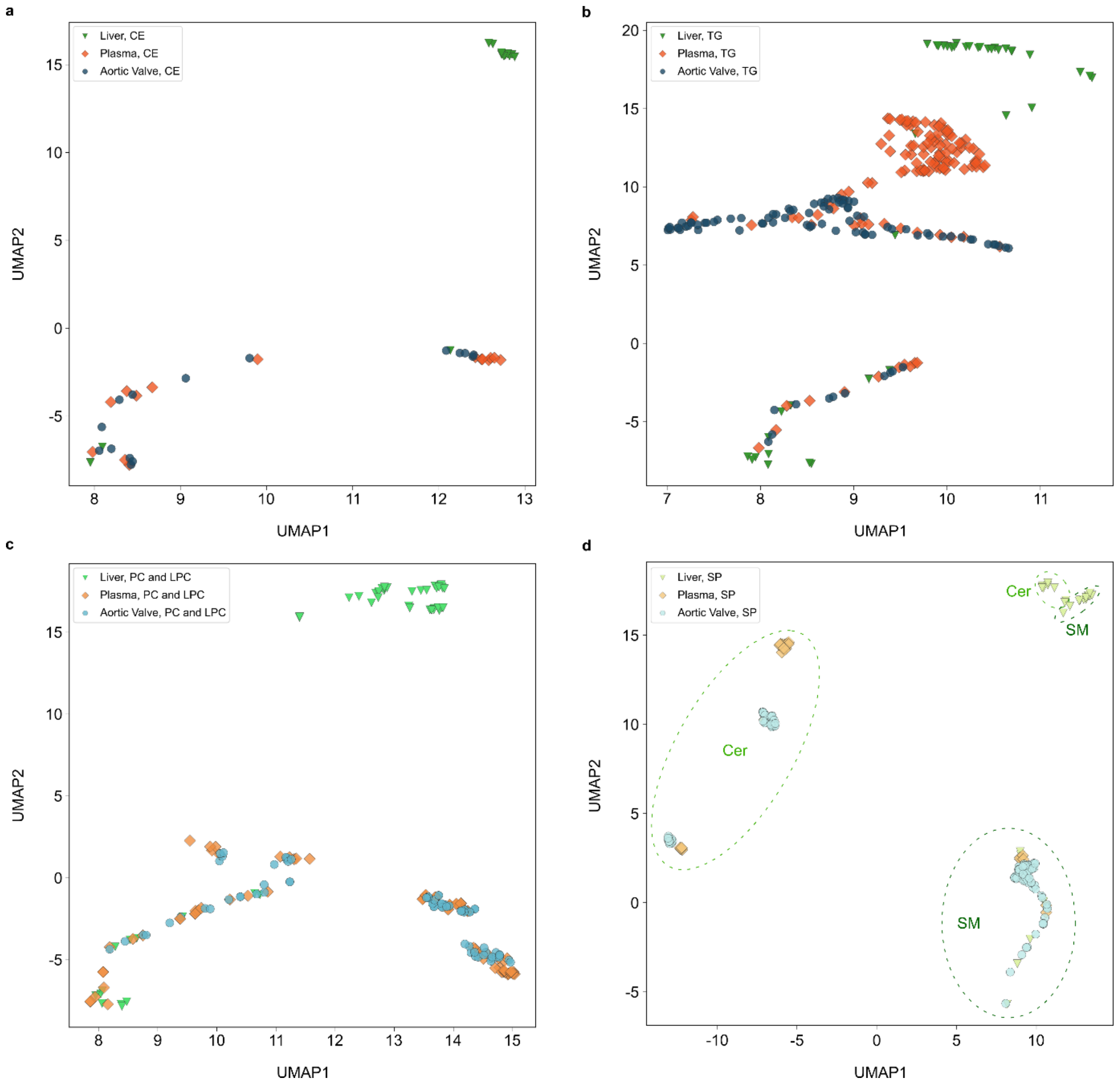

Supplementary Fig. 1 | Uniform Manifold Approximation and Projection (UMAP) topological comparison of human AV, blood plasma and liver lipidomes depicted by lipid classes. UMAP plots are depicted separately for CE (**a**), TG (**b**), PC and LPC (**c**) and Sphingolipids (SP, **d**). Source data are provided as a Source Data file.

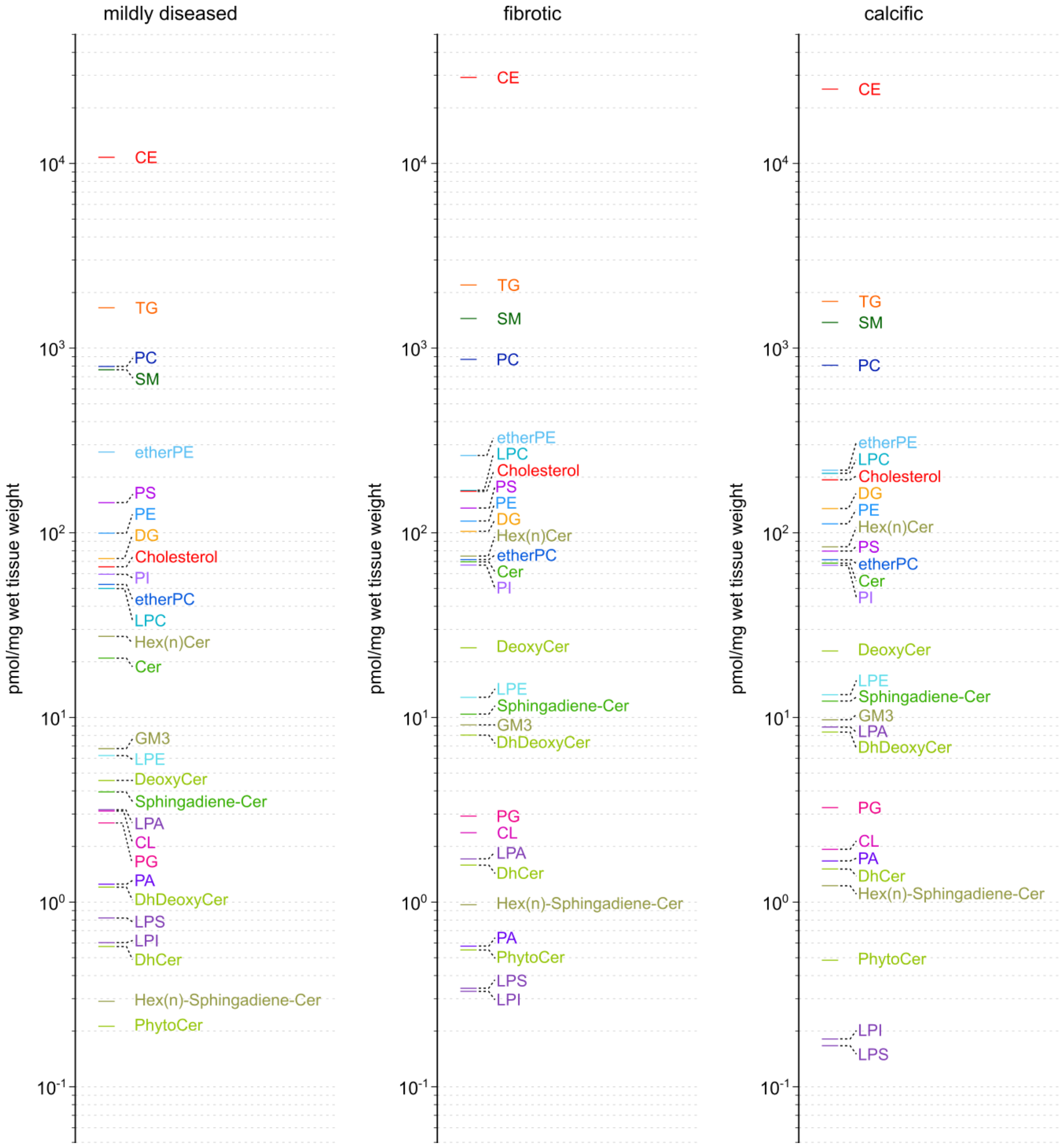

Supplementary Fig. 2 | Quantitative distribution of lipid classes in mildly diseased, fibrotic and calcific human TAV of elderly individuals. Bold lines mark total lipid class concentration. Source data are provided as a Source Data file.

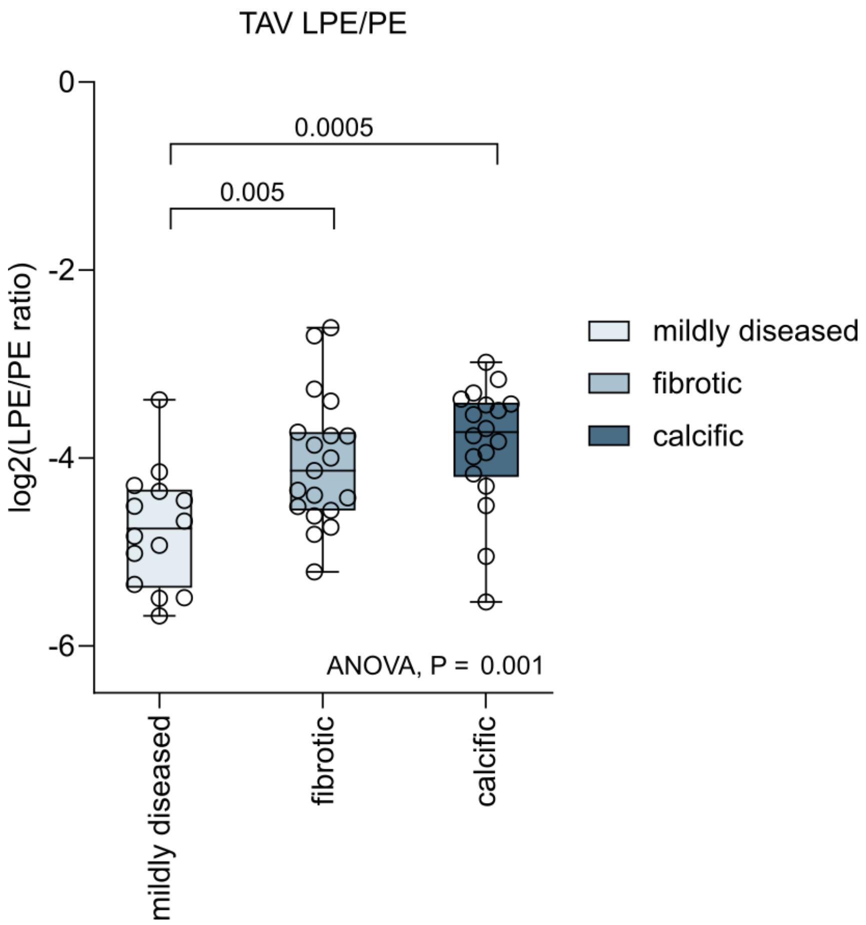

Supplementary Fig. 3 | Total LPE to PE ratios in mildly diseased, fibrotic and calcific AV sections. P values were calculated by two-sided T-test or by ordinary one-way ANOVA across all groups. Boxplot elements represent centerline, mean; box limits, 25% and 75% quartiles; vertical lines connect minimum and maximum values. Dots represent biologically independent samples (n=21). Color-coding indicates pathophysiological state: light blue, mildly diseased; blue, fibrotic; dark blue, calcific. Source data are provided as a Source Data file.

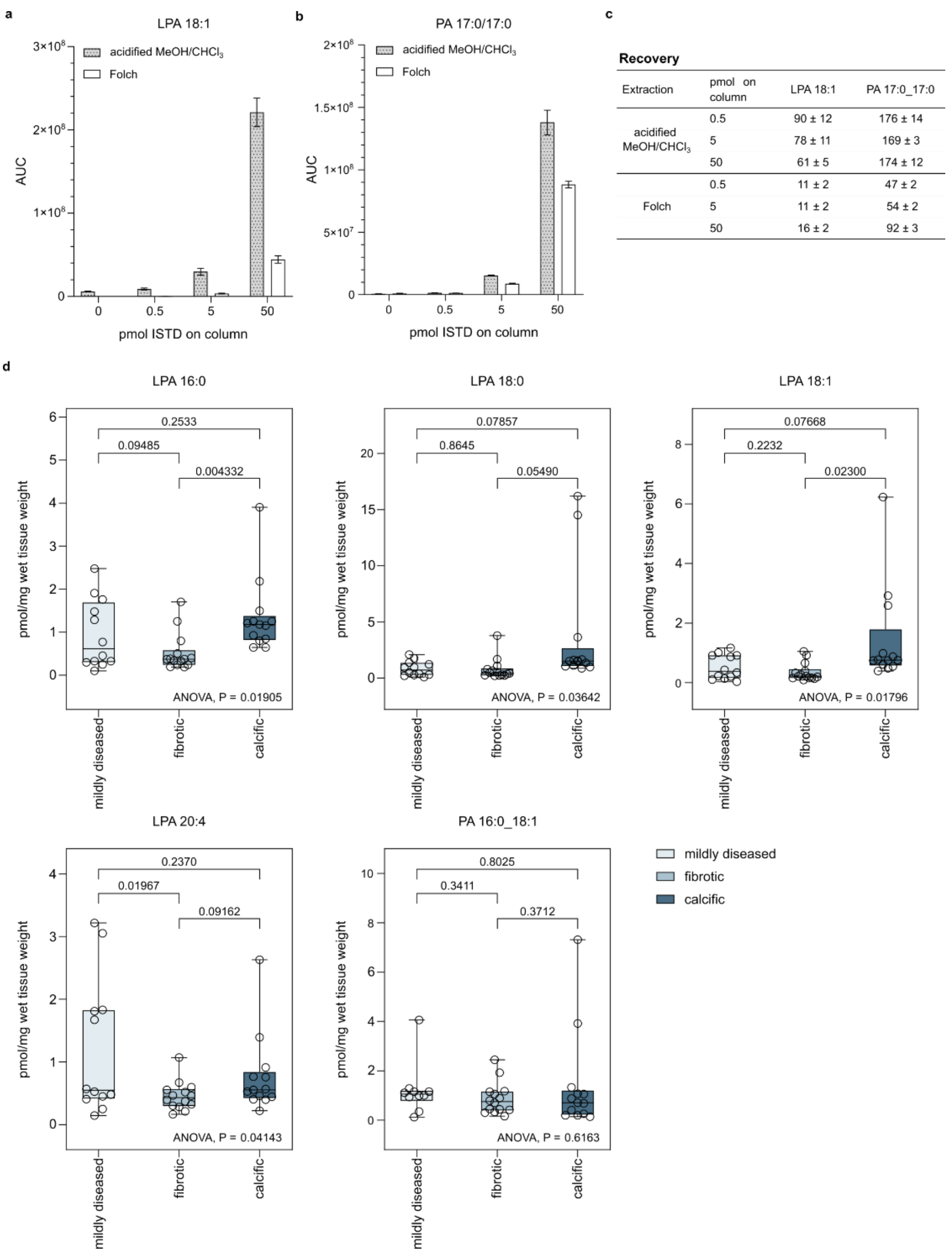

Recovery

Extraction	pmol on column	LPA 18:1	PA 17:0_17:0
acidified $MeOH/CHCl_3$	0.5	90 ± 12	176 ± 14
	5	78 ± 11	169 ± 3
	50	61 ± 5	174 ± 12
Folch	0.5	11 ± 2	47 ± 2
	5	11 ± 2	54 ± 2
	50	16 ± 2	92 ± 3

Supplementary Fig. 4 | Phosphatidic acid (PA) and Lysophosphatidic acid (LPA) are only extracted sufficiently by acidified MeOH/CHCl3 lipid extraction. a-b, Area under curve values for different initial LPA 18:1 (**a**) or PA 17:0/17:0 (**b**) concentrations, either extracted by acidified MeOH/CHCl3 or Folch extraction. **c,** Recovery rates for LPA 18:1 and PA 17:0/17:0, either extracted by acidified MeOH/CHCl3 or Folch extraction. **d,** Concentration values for all identified PA and LPA lipid species in mildly diseased, fibrotic and calcific AV sections. P values were calculated by two-sided T-test or by ordinary one-way ANOVA across all groups. Boxplot elements represent centerline, mean; box limits, 25% and 75% quartiles; vertical lines connect minimum and maximum values. Dots represent biologically independent samples (n=14). Color-coding indicates pathophysiological state: light blue, mildly diseased; blue, fibrotic; dark blue, calcific. Source data are provided as a Source Data file.

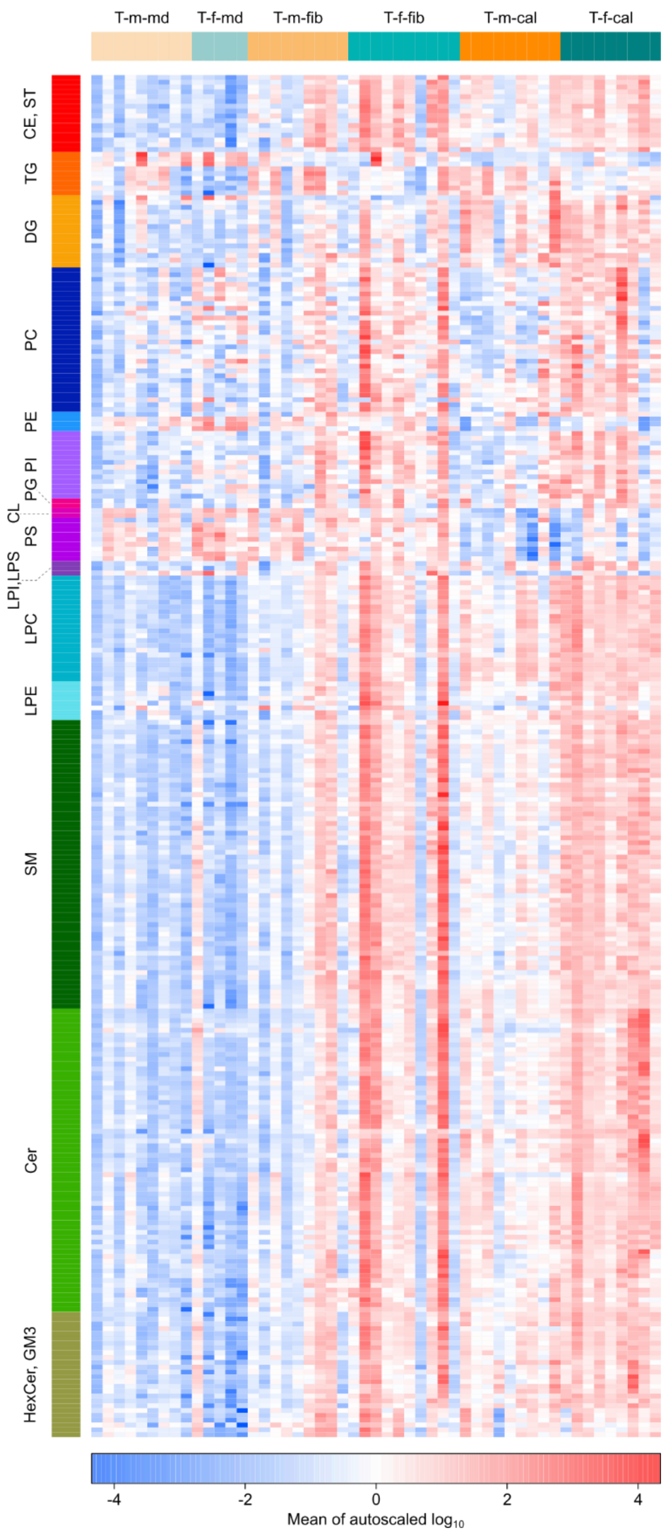

Supplementary Fig. 5 | Heatmap illustrates TAV lipidomes of female and male individuals across different pathophysiological stages of FCAVD. Heatmap only includes lipid species significantly regulated between female versus male individuals (n=21) across different pathophysiological stages, including mildly diseased, fibrotic and calcific in TAV tissue sections determined one-way ANOVA & post-hoc ($P < 0.05$). Source data are provided as a Source Data file.

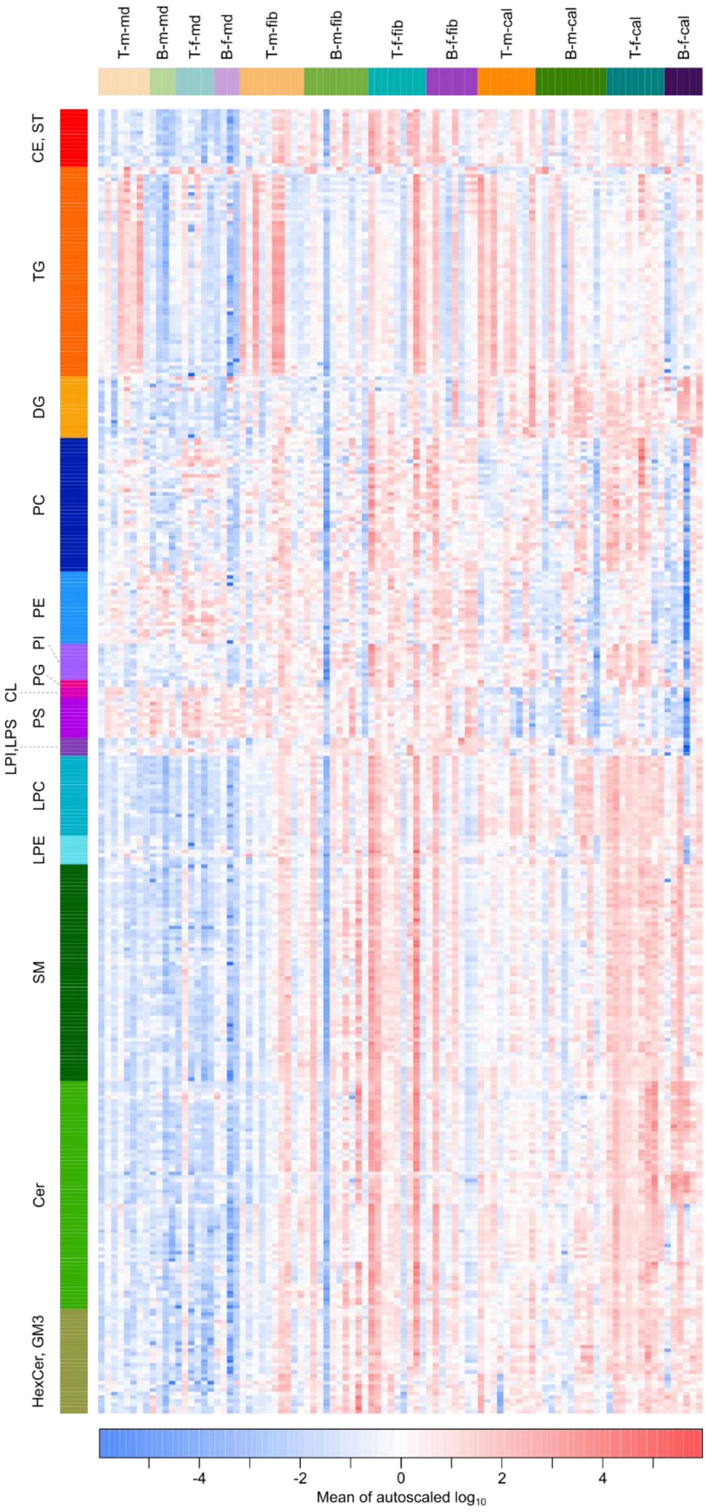

Supplementary Fig. 6 | Heatmap illustrates TAV and BAV lipidomes of female and male individuals across different pathophysiological stages of FCAVD. Heatmap only includes lipid species significantly regulated between female versus male individuals (n=41) across different pathophysiological stages, including mildly diseased, fibrotic and calcific in TAV and BAV tissue sections determined one-way ANOVA & post-hoc ($P < 0.05$). Source data are provided as a Source Data file.

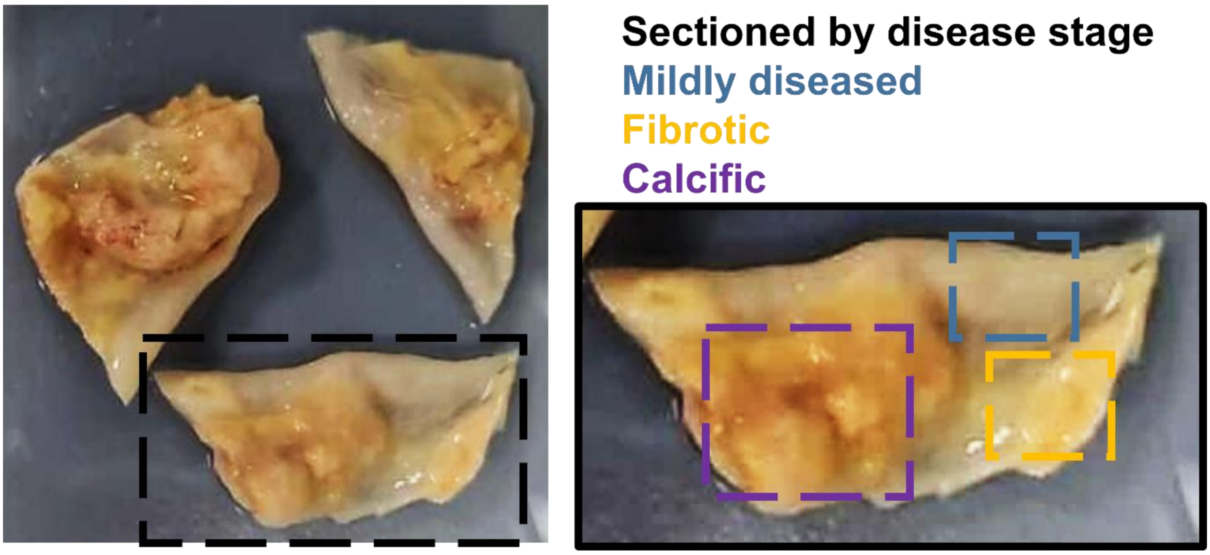

Supplementary Fig. 7 | Visual representation of aortic valves sectioning. AV were macroscopically inspected and dissected into mildly diseased (blue area), fibrotic (yellow area) and calcific (violet area) sections.

	Identification		Calibration		Quantification	
	**a) Untargeted lipidomics for human AV lipids annotation**		**b) Design of the AV-tailored mixture of ISTD for quantification**	**c) Assessment of PA and LPA recovery**	**d) Untargeted lipidomics for human AV lipids quantification**	**e) Targeted lipidomics for human AV lipids quantification**
Sample type	pooled AV sample		pooled AV sample and ISTD		I. individual samples with added one-point ISTD mixture<br>II. batch quality control (BQC) (pooled from AV samples)<br>III. total quality control (tQC) pooled from individual AV samples	I. individual samples with added one-point ISTD mixture<br>II. batch quality control (BQC) (pooled from AV samples)<br>III. total quality control (tQC) pooled from individual AV samples
Extraction	Folch	Folch	Folch	Folch, acidified MeOH/$CHCl_3$	Folch	Folch, acidified MeOH/$CHCl_3$
LC	RPC30 method 1 (34 min gradient)	RPC30 method 2 (57 min gradient)	RPC30 method 1 (34 min gradient)	RPC30 method 1 (34 min gradient)	RPC30 method 3 (47 min gradient)	RPC18 method 4 (15 min gradient)
MS	DDA (Q Exactive Plus Hybrid Quadrupole Orbitrap)	DDA (Exploris 240 Hybrid Quadrupole Orbitrap)	MS1 (Q Exactive Plus Hybrid Quadrupole Orbitrap)	DDA (Exploris 240 Hybrid Quadrupole Orbitrap)	MS1 (Exploris 240 Hybrid Quadrupole Orbitrap)	SRM (Altis Plus Triple Quadrupole)

Supplementary Fig. 8 | Schematic overview of the experimental design used for the lipidomics analysis. Sample types, extraction methods, LC and MS acquisition types listed for identification (a), calibration (b and c), and quantification (d and e) experiments. DDA – data-dependent acquisition; ISDT – internal standards; LPA – lysophosphatidic acid, PA - phosphatidic acid; SRM – single reaction monitoring.

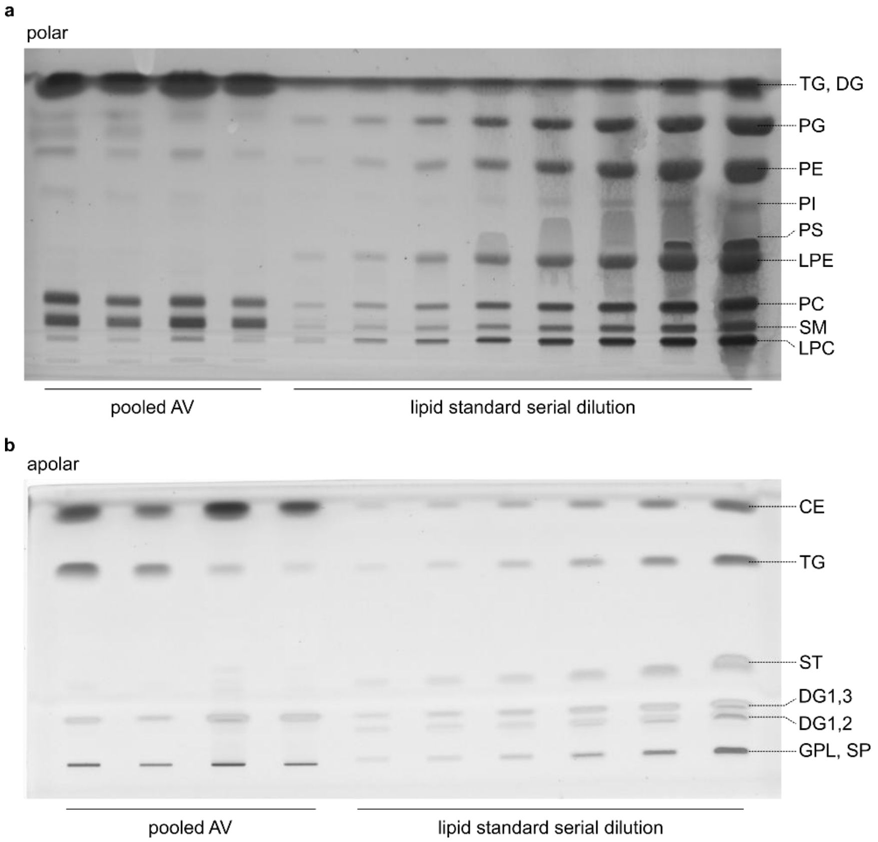

Supplementary Fig. 9 | Quantitative high-performance thin-layer chromatograms (qHPTLC) for lipid class specific quantification of aortic valve lipids. a, The polar plate was developed using chloroform/ethanol/trimethylamine/water (5:5:5:1, v/v/v/v). **b**, The apolar plate was developed using hexane/diethylether/acetic acid (85:15:1, v/v/v/v).

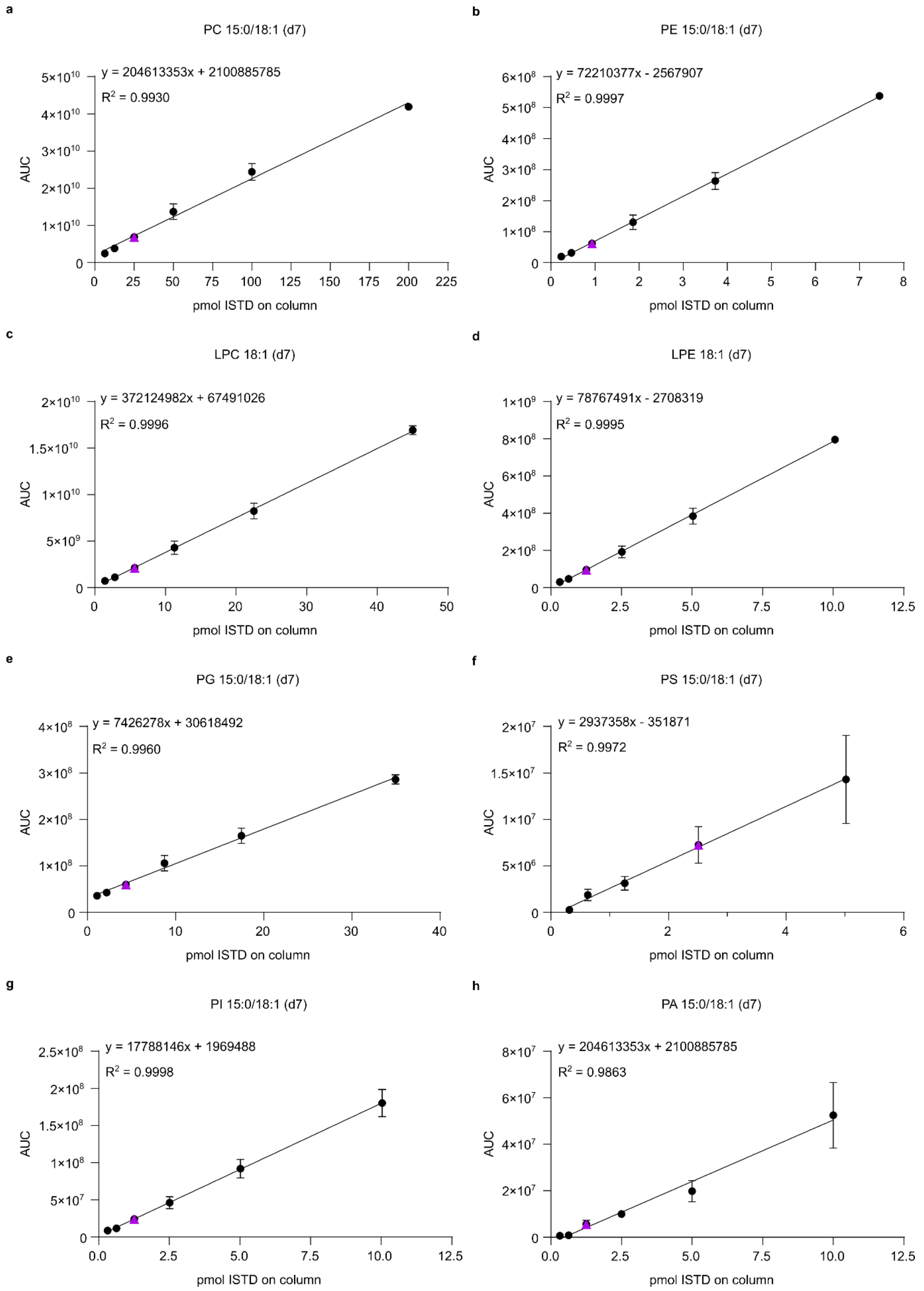
a
PC 15:0/18:1 (d7)
y = 204613353x + 2100885785
R2 = 0.9930
AUC
pmol ISTD on column
b
PE 15:0/18:1 (d7)
y = 72210377x - 2567907
R2 = 0.9997
AUC
pmol ISTD on column
c
LPC 18:1 (d7)
y = 372124982x + 67491026
R2 = 0.9996
AUC
pmol ISTD on column
d
LPE 18:1 (d7)
y = 78767491x - 2708319
R2 = 0.9995
AUC
pmol ISTD on column
e
PG 15:0/18:1 (d7)
y = 7426278x + 30618492
R2 = 0.9960
AUC
pmol ISTD on column
f
PS 15:0/18:1 (d7)
y = 2937358x - 351871
R2 = 0.9972
AUC
pmol ISTD on column
g
PI 15:0/18:1 (d7)
y = 17788146x + 1969488
R2 = 0.9998
AUC
pmol ISTD on column
h
PA 15:0/18:1 (d7)
y = 204613353x + 2100885785
R2 = 0.9863
AUC
pmol ISTD on column

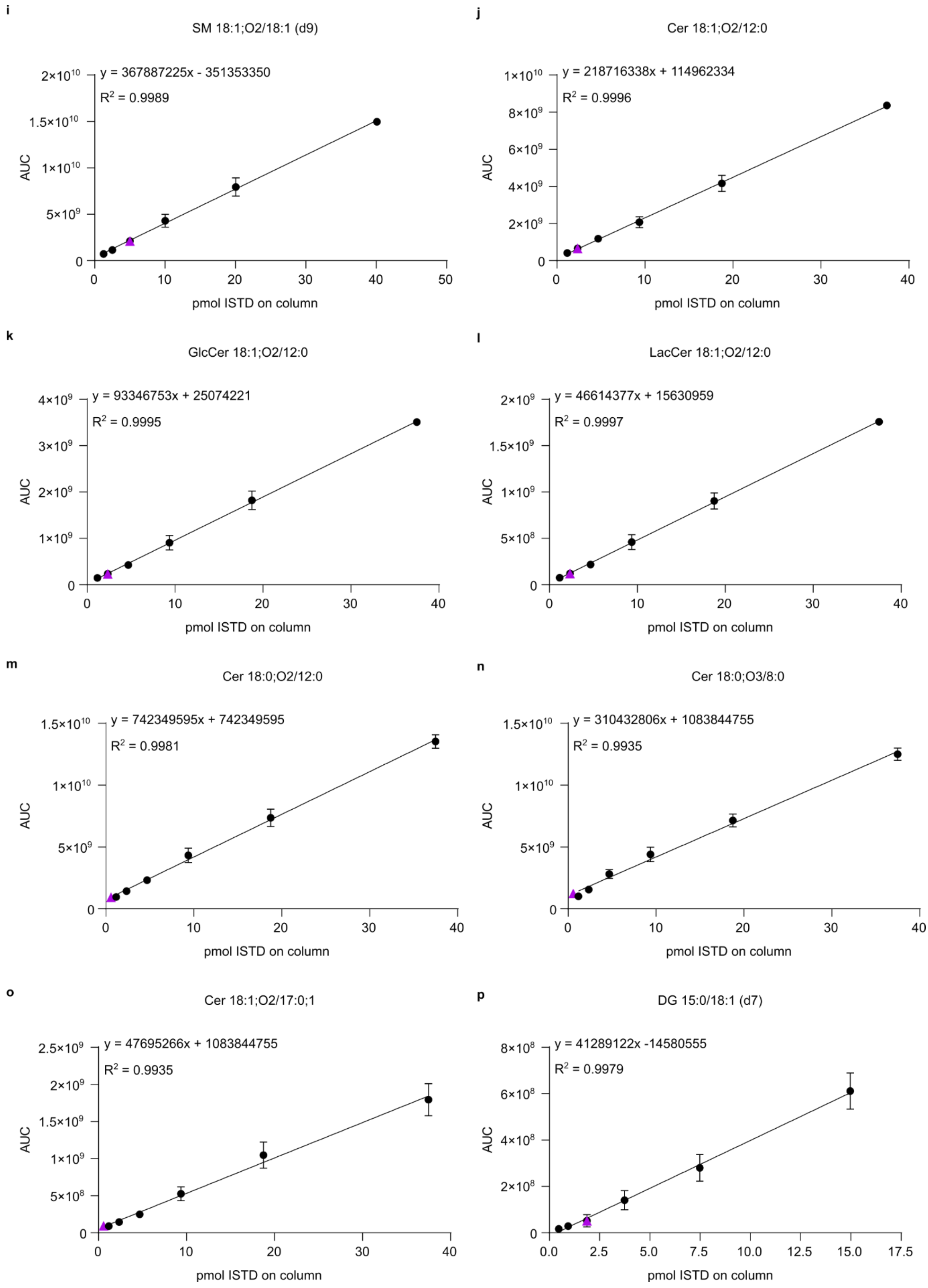
i
SM 18:1;O2/18:1 (d9)
y = 367887225x - 351353350
R2 = 0.9989
AUC
pmol ISTD on column
j
Cer 18:1;O2/12:0
y = 218716338x + 114962334
R2 = 0.9996
AUC
pmol ISTD on column
k
GlcCer 18:1;O2/12:0
y = 93346753x + 25074221
R2 = 0.9995
AUC
pmol ISTD on column
l
LacCer 18:1;O2/12:0
y = 46614377x + 15630959
R2 = 0.9997
AUC
pmol ISTD on column
m
Cer 18:0;O2/12:0
y = 742349595x + 742349595
R2 = 0.9981
AUC
pmol ISTD on column
n
Cer 18:0;O3/8:0
y = 310432806x + 1083844755
R2 = 0.9935
AUC
pmol ISTD on column
o
Cer 18:1;O2/17:0;1
y = 47695266x + 1083844755
R2 = 0.9935
AUC
pmol ISTD on column
p
DG 15:0/18:1 (d7)
y = 41289122x -14580555
R2 = 0.9979
AUC
pmol ISTD on column

q

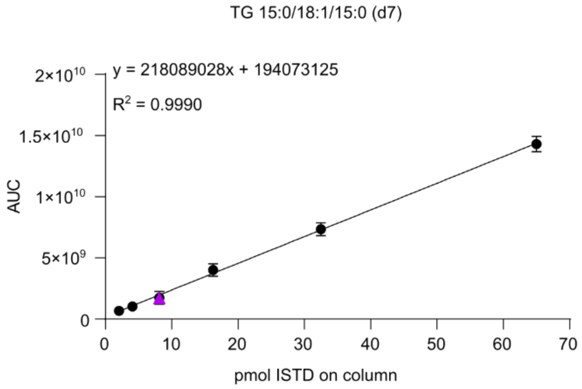
TG 15:0/18:1/15:0 (d7)
y = 218089028x + 194073125
R2 = 0.9990
2×10^10
1.5×10^10
1×10^10
5×10^9
0
AUC
0
10
20
30
40
50
60
70
pmol ISTD on column

r

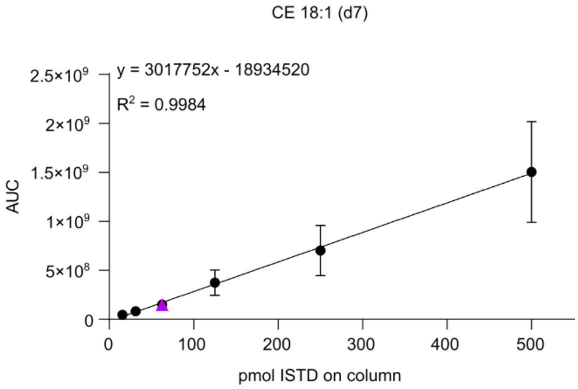
CE 18:1 (d7)
y = 3017752x - 18934520
R2 = 0.9984
2.5×10^9
2×10^9
1.5×10^9
1×10^9
5×10^8
0
AUC
0
100
200
300
400
500
pmol ISTD on column

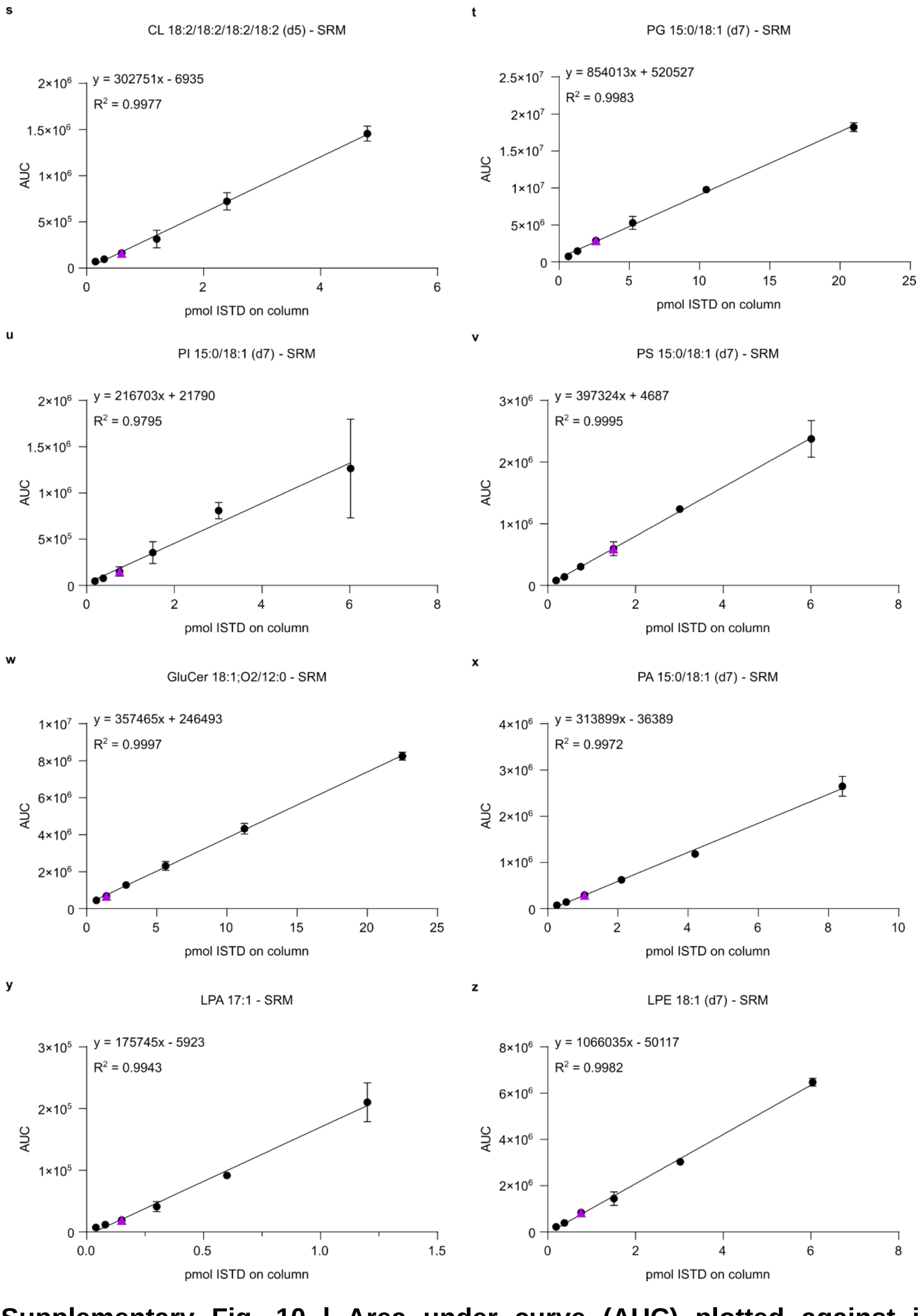

Supplementary Fig. 10 | Area under curve (AUC) plotted against internal standard (ISTD) concentration in order to establish calibration curves for different AV lipid class standards. Each calibration point was determined in three independent replicates of pooled AV tissue powder. **a - r,** Data used for the calibration curves was acquired using a Q Exactive Plus Hybrid Quadrupole Orbitrap mass spectrometer in full scan mode. **s – z,** Data used for the calibration curves was acquired using a TSQ Altis Plus Triple Quadrupole mass spectrometer in selected reaction monitoring (SRM). Source data are provided as a Source Data file.

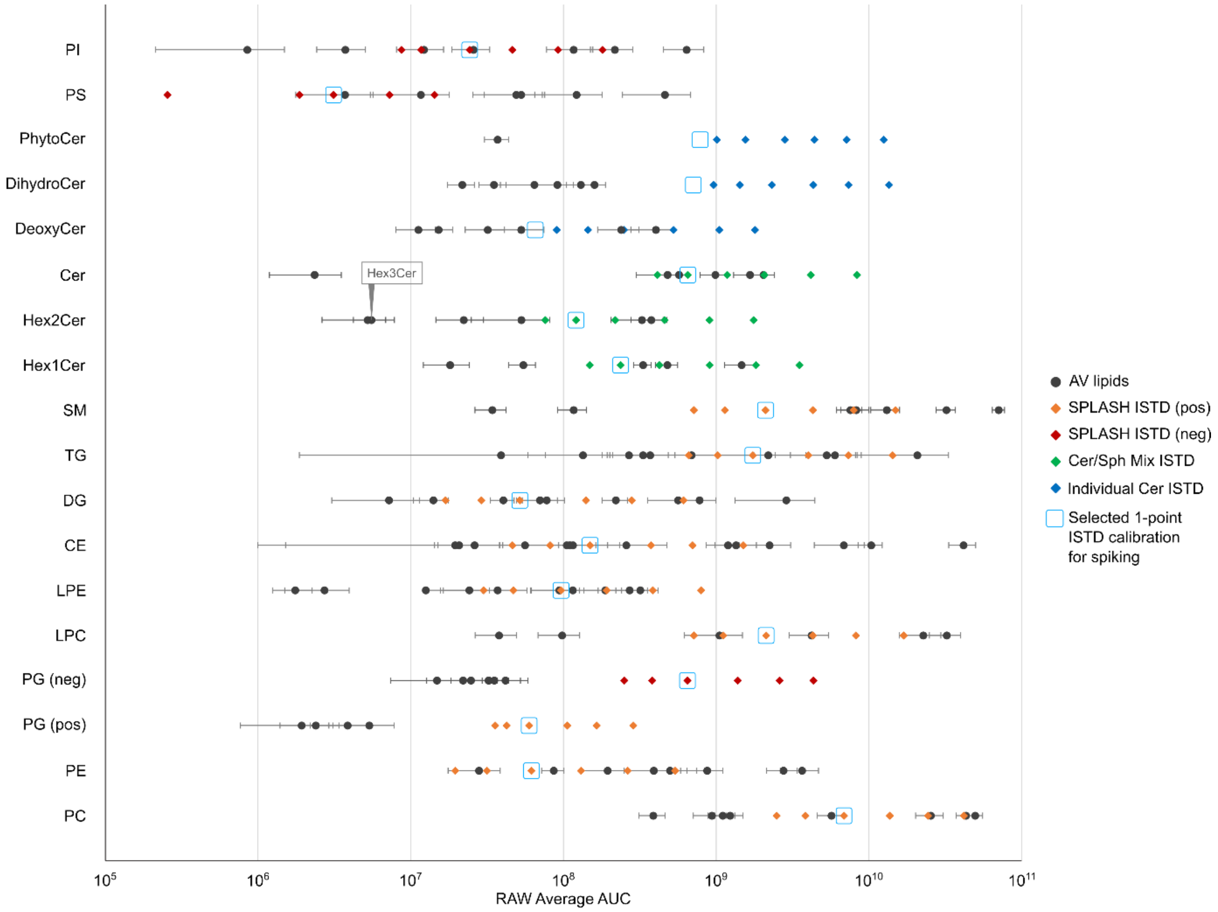

Supplementary Fig. 11 | Concentration alignment of internal standard (ISTD) mixtures and endogenous aortic valve (AV) lipids. Peak areas of five to nine endogenous lipids per subclass, representing some of the most, middle and the least abundant species of the class, were plotted alongside the peak areas of the corresponding lipid class ISTD in order to select the ideal ISTD amounts for each class (blue rectangle). Mean peak areas and standard deviations (SD) for lipid class standards and representative endogenous AV lipids per subclass were determined in three independent replicates of pooled AV tissue powder. Source data are provided as a Source Data file.

Supplementary Note 1 | Lipidomics Minimal Reporting Checklist with details on the AV lipid annotation and quantification using untargeted lipidomics workflow.

Contents of Report

Created by https://lipidomicstandards.org, version v2.4.0

Separation Workflow 1
Overall study design 1
Lipid extraction 1
Analytical platform 1
Quality control 2
Method qualification and validation 2
Reporting 2

Sample Descriptions 2
Aortic valve tissue / Human / Tissues (e.g., liver, heart, brain) 2

Lipid Class Descriptions 3
1) CE[M+NH4]+ / Lipid identification 3
1) CE[M+NH4]+ / Lipid quantification 3
2) FC[M-H2O+H]+ / Lipid identification 4
2) FC[M-H2O+H]+ / Lipid quantification 4
3) TG[M+NH4]+ / Lipid identification 5
3) TG[M+NH4]+ / Lipid quantification 6
4) DG[M+NH4]+ / Lipid identification 6
4) DG[M+NH4]+ / Lipid quantification 7
5) PC[M+HCOO]- / Lipid identification 7
5) PC[M+HCOO]- / Lipid quantification 8
6) PE[M-H]- / Lipid identification 8
6) PE[M-H]- / Lipid quantification 9
7) PA[M-H]- / Lipid identification 10
7) PA[M-H]- / Lipid quantification 10
8) PS[M-H]- / Lipid identification 11
8) PS[M-H]- / Lipid quantification 11
9) PI[M-H]- / Lipid identification 12
9) PI[M-H]- / Lipid quantification 12
10) PG[M-H]- / Lipid identification 13
10) PG[M-H]- / Lipid quantification 13
11) CL[M-H]- / Lipid identification 14
11) CL[M-H]- / Lipid quantification 14
12) LPC[M+HCOO]- / Lipid identification 15
12) LPC[M+HCOO]- / Lipid quantification 15
13) LPE[M-H]- / Lipid identification 16
13) LPE[M-H]- / Lipid quantification 16
14) LPA[M-H]- / Lipid identification 17
14) LPA[M-H]- / Lipid quantification 17
15) LPI[M-H]- / Lipid identification 18
15) LPI[M-H]- / Lipid quantification 18
16) LPS[M-H]- / Lipid identification 19
16) LPS[M-H]- / Lipid quantification 19
17) SM[M+H]+ / Lipid identification 20
17) SM[M+H]+ / Lipid quantification 20
18) Cer[M+H]+ / Lipid identification 21
18) Cer[M+H]+ / Lipid quantification 22
19) HexCer[M+H]+ / Lipid identification 23
19) HexCer[M+H]+ / Lipid quantification 24
20) Hex2Cer[M+H]+ / Lipid identification 25
20) Hex2Cer[M+H]+ / Lipid quantification 26
21) Hex3Cer[M+H]+ / Lipid identification 26
21) Hex3Cer[M+H]+ / Lipid quantification 27
22) GM3[M-H]- / Lipid identification 27
22) GM3[M-H]- / Lipid quantification 28

Separation Workflow

Overall study design

Title of the study	Sex-specific lipidomic signatures in aortic valve disease reflect differential fibro-calcific progression (untargeted)		
Document creation date	12/20/2024	Corresponding Email	maria.fedorova@tu-dresden.de
Principal investigator	Maria Fedorova	Is the workflow targeted or untargeted?	Untargeted
Institution	Technical University Dresden, Germany	Clinical	No

Lipid extraction

Extraction method	2-phase system	Were internal standards added prior extraction?	Yes
pH adjustment	None	Special conditions	All solvents used for extraction were supplemented with 0.1 % (w/v) BHT. All extraction steps were performed on ice.
2-phase system	Folch	Derivatization	-

Analytical platform

Ionizaton additives	Ammonium formate, Formic acid	Resolution at m/z 200 at MS1	140000
Number of separation dimensions	One dimension	Mass accuracy in ppm at MS1	3
Separation type 1	LC	Recording mode of raw data at MS1	Profile mode
Separation mode 1 (liquid)	RP	Mass window for precursor ion isolation (in Da total isolation window)	1.2
Detector	Mass spectrometer	Mass resolution for detected ion at MS2	High resolution
MS type	Orbitrap	Resolution at m/z 200 at MS2	17500
MS vendor	Thermo	Mass accuracy in ppm at MS2	10
Ion source	ESI	Recording mode of raw data at MS2	Profile mode
MS Level	MS1, MS2	Was/Were additional dimension/techniques used	No
Mass resolution for detected ion at MS1	High resolution		

Quality control

Blanks	Yes	Quality control	Yes
Type of Blanks	Solvent blank, Internal standard blank	Type of QC sample	Sample pool

Method qualification and validation

Method validation	Yes	Precision	No
Lipid recovery	No	Accuracy	No
Dynamic quantification range	Yes	Guidelines followed	None
Limit of quantitation (LOQ)/Limit of detection (LOD)	Yes		

Reporting

Are reported raw data uploaded into repository?	Yes	Summary data	Quantification and identification data
Link to repository / ID to entry	doi: 10.21228/M8G24F	Raw data upload	Yes
Are metadata available?	Yes	Additional comments	-

Sample Descriptions

Aortic valve tissue / Human / Tissues (e.g., liver, heart, brain)

Perfusion	No	Additives	None
Storage and collection conditions	Available	Were samples stored under inert gas?	No
Provided preanalytical information	Freeze-thaw cycles	Additional preservation methods	No
Temperature handling original sample	Room temperature	Biobank samples	No
Instant sample preparation	No	Sample homogenization	Yes
Storage temperature	-80 °C	Sample homogenization solvent	liquid nitrogen
Freeze-thaw cycles	1		

Lipid Class Descriptions

1) CE[M+NH4]+ / Lipid identification

Lipid class	CE	Did you presume assumptions for identification?	No
MS Level for identification	MS1, MS2	Check on:	Isomeric overlap, Isobaric overlap, In-source fragmentation
Identification level	Molecular species level	Limit of detection	Signal theshold
Polarity mode	Positive	RT verified by standard	Yes
Type of positive (precursor)ion	[M+NH4]+	Separation of isobaric/isomeric interferece confirmed	No
Fragments for identification	Fragment name: -FA (+OH)	Model for separation prediction	No
Isotope correction at MS1	No	Additional dimension/techniques	-
Isotope correction at MS2	No	Lipid Identification Software	manually
MS1 verified by standard	Yes	Data manipulation	-
MS2 verified by standard	Yes	Nomenclature for intact lipid molecule	Yes
Background check at MS1	Yes	Nomenclature for fragment ions	N/A
Background check at MS2	Yes	Further identification remarks	Proposed identifications were further validated by plotting the retention time of lipid species against their Kendrick mass defect by hydrogen and lipid species not following expected trendlines were excluded.

1) CE[M+NH4]+ / Lipid quantification

Quantitative	Yes	Limit of quantification	Linear regression analysis was applied by plotting the calculated concentrations against their AUC value to identify and exclude possible outliers and features showing non-linear behaviour.
MS Level for quantification	MS1	Normalization to reference	Yes
Internal lipid standard(s) MS1	Internal standard: CE 18:1 (d7); Endogenous subclass: CE	Lipid Quantification Software	Skyline
Type of quantification	Internal standard amount	Batch correction	No
Response correction	No	Further quantification remarks	Individual lipid species concentrations were normalized to the wet tissue weight.
Type I isotope correction	Yes		

2) FC[M-H2O+H]+ / Lipid identification

Lipid class	FC	Limit of detection	Signal theshold
MS Level for identification	MS1	RT verified by standard	Yes
Identification level	Species level	Separation of isobaric/isomeric interferece confirmed	No
Polarity mode	Positive	Model for separation prediction	No
Type of positive (precursor)ion	[M-H2O+H]+	Additional dimension/techniques	-
Isotope correction at MS1	No	Lipid Identification Software	manually
MS1 verified by standard	Yes	Data manipulation	-
Background check at MS1	Yes	Nomenclature for intact lipid molecule	Yes
Did you presume assumptions for identification?	No	Further identification remarks	-
Check on:	Isomeric overlap, Isobaric overlap, In-source fragmentation		

2) FC[M-H2O+H]+ / Lipid quantification

Quantitative	Yes	Limit of quantification	Linear regression analysis was applied by plotting the calculated concentrations against their AUC value to identify and exclude possible outliers and features showing non-linear behaviour.
MS Level for quantification	MS1	Normalization to reference	Yes
Internal lipid standard(s) MS1		Lipid Quantification Software	Skyline
Type of quantification	Internal standard amount	Batch correction	No
Response correction	No	Further quantification remarks	Individual lipid species concentrations were normalized to the wet tissue weight.
Type I isotope correction	Yes		

Internal lipid standard(s) MS1:

Internal standard	Endogenous subclass
Cholesterol (d7)	FC

3) TG[M+NH4]+ / Lipid identification

Lipid class	TG	Did you presume assumptions for identification?	No
MS Level for identification	MS1, MS2	Check on:	Isomeric overlap, Isobaric overlap, In-source fragmentation
Identification level	Molecular species level	Limit of detection	Signal theshold
Polarity mode	Positive	RT verified by standard	Yes
Type of positive (precursor)ion	[M+NH4]+	Separation of isobaric/isomeric interferece confirmed	No
Fragments for identification		Model for separation prediction	No

Fragment name
FA1-(H2O)
FA2-(H2O)
FA3-(H2O)
-FA1(+HO)-(NH3)
-FA2(+HO)-(NH3)
-FA3(+HO)-(NH3)
MG1(+HO)-(NH3)
MG2(+HO)-(NH3)
MG3(+HO)-(NH3)

Isotope correction at MS1	No	Additional dimension/techniques	-
Isotope correction at MS2	No	Lipid Identification Software	LipidHunter
MS1 verified by standard	Yes	Data manipulation	-
MS2 verified by standard	Yes	Nomenclature for intact lipid molecule	Yes
Background check at MS1	Yes	Nomenclature for fragment ions	N/A
Background check at MS2	Yes	Further identification remarks	Proposed identifications were further validated by plotting the retention time of lipid species against their Kendrick mass defect by hydrogen and lipid species not following expected trendlines were excluded.

3) TG[M+NH4]+ / Lipid quantification

Quantitative	Yes	Limit of quantification	Linear regression analysis was applied by plotting the calculated concentrations against their AUC value to identify and exclude possible outliers and features showing non-linear behaviour.
MS Level for quantification	MS1	Normalization to reference	Yes
Internal lipid standard(s) MS1		Lipid Quantification Software	Skyline

Internal standard	Endogenous subclass
TG 15:0/18:1/15:0 (d7)	TG

Type of quantification	Internal standard amount	Batch correction	No
Response correction	No	Further quantification remarks	Individual lipid species concentrations were normalized to the wet tissue weight.
Type I isotope correction	Yes		

4) DG[M+NH4]+ / Lipid identification

Lipid class	DG	Did you presume assumptions for identification?	No
MS Level for identification	MS1, MS2	Check on:	Isomeric overlap, Isobaric overlap, In-source fragmentation
Identification level	Molecular species level	Limit of detection	Signal theshold
Polarity mode	Positive	RT verified by standard	Yes
Type of positive (precursor)ion	[M+NH4]+	Separation of isobaric/isomeric interferece confirmed	No
Fragments for identification		Model for separation prediction	No

Fragment name
-NH3
-NH3-H2O
-FA1(+HO)-(NH3)
-FA2(+HO)-(NH3)
FA1(-HO)
FA1(-HO)

Isotope correction at MS1	No	Additional dimension/techniques	-
Isotope correction at MS2	No	Lipid Identification Software	LipidHunter
MS1 verified by standard	Yes	Data manipulation	-
MS2 verified by standard	Yes	Nomenclature for intact lipid molecule	Yes
Background check at MS1	Yes	Nomenclature for fragment ions	N/A
Background check at MS2	Yes	Further identification remarks	Proposed identifications were further validated by plotting the retention time of lipid species against their Kendrick mass defect by hydrogen and lipid species not following expected trendlines were excluded.

4) DG[M+NH4]+ / Lipid quantification

Quantitative	Yes	Limit of quantification	Linear regression analysis was applied by plotting the calculated concentrations against their AUC value to identify and exclude possible outliers and features showing non-linear behaviour.
MS Level for quantification	MS1	Normalization to reference	Yes
Internal lipid standard(s) MS1		Lipid Quantification Software	Skyline
Type of quantification	Internal standard amount	Batch correction	No
Response correction	No	Further quantification remarks	Individual lipid species concentrations were normalized to the wet tissue weight.
Type I isotope correction	Yes		

Internal standard	Endogenous subclass
DG 15:0/18:1 (d7)	DG

5) PC[M+HCOO]- / Lipid identification

Lipid class	PC	Did you presume assumptions for identification?	No
MS Level for identification	MS1, MS2	Check on:	Isomeric overlap, Isobaric overlap, In-source fragmentation
Identification level	Molecular species level	Limit of detection	Signal theshold
Polarity mode	Negative	RT verified by standard	Yes
Type of negative (precursor)ion	[M+HCOO]-	Separation of isobaric/isomeric interferece confirmed	No
Fragments for identification		Model for separation prediction	No
Isotope correction at MS1	No	Additional dimension/techniques	-
Isotope correction at MS2	No	Lipid Identification Software	LipidHunter
MS1 verified by standard	Yes	Data manipulation	-
MS2 verified by standard	Yes	Nomenclature for intact lipid molecule	Yes
Background check at MS1	Yes	Nomenclature for fragment ions	N/A
Background check at MS2	Yes	Further identification remarks	Proposed identifications were further validated by plotting the retention time of lipid species against their Kendrick mass defect by hydrogen and lipid species not following expected trendlines were excluded.

Fragment name
-(CH3+HCOO)
-FA1(-H)-(CH3+HCOO)
-FA2(-H)-(CH3+HCOO)
FA1(-H)
FA2(-H)
HG(PC,168)

5) PC[M+HCOO]- / Lipid quantification

Quantitative	Yes	Limit of quantification	Linear regression analysis was applied by plotting the calculated concentrations against their AUC value to identify and exclude possible outliers and features showing non-linear behaviour.
MS Level for quantification	MS1	Normalization to reference	Yes
Internal lipid standard(s) MS1		Lipid Quantification Software	Skyline

Internal standard	Endogenous subclass
PC 15:0/18:1 (d7)	PC

Type of quantification	Internal standard amount	Batch correction	No
Response correction	No	Further quantification remarks	Individual lipid species concentrations were normalized to the wet tissue weight.
Type I isotope correction	Yes		

6) PE[M-H]- / Lipid identification

Lipid class	PE	Did you presume assumptions for identification?	No
MS Level for identification	MS1, MS2	Check on:	Isomeric overlap, Isobaric overlap, In-source fragmentation
Identification level	Molecular species level	Limit of detection	Signal theshold
Polarity mode	Negative	RT verified by standard	Yes
Type of negative (precursor)ion	[M-H]-	Separation of isobaric/isomeric interferece confirmed	No
Fragments for identification		Model for separation prediction	No

Fragment name
-FA1
-FA2
FA1(-H)
FA2(-H)
HG(PE,140)

Isotope correction at MS1	No	Additional dimension/techniques	-
Isotope correction at MS2	No	Lipid Identification Software	LipidHunter
MS1 verified by standard	Yes	Data manipulation	-
MS2 verified by standard	Yes	Nomenclature for intact lipid molecule	Yes
Background check at MS1	Yes	Nomenclature for fragment ions	N/A
Background check at MS2	Yes	Further identification remarks	Proposed identifications were further validated by plotting the retention time of lipid species against their Kendrick mass defect by hydrogen and lipid species not following expected trendlines were excluded.

6) PE[M-H]- / Lipid quantification

Quantitative	Yes	Limit of quantification	Linear regression analysis was applied by plotting the calculated concentrations against their AUC value to identify and exclude possible outliers and features showing non-linear behaviour.
MS Level for quantification	MS1	Normalization to reference	Yes
Internal lipid standard(s) MS1		Lipid Quantification Software	Skyline
Type of quantification	Internal standard amount	Batch correction	No
Response correction	No	Further quantification remarks	Individual lipid species concentrations were normalized to the wet tissue weight.
Type I isotope correction	Yes		

Internal standard	Endogenous subclass
PE 15:0/18:1 (d7)	PE

7) PA[M-H]- / Lipid identification

Lipid class	PA	Which assumptions were presumed?	It was checked that the identified PA is of endogeneous origin and not the result of in-source fragmentation of other PL.
MS Level for identification	MS1, MS2	Check on:	Isomeric overlap, Isobaric overlap, In-source fragmentation
Identification level	Molecular species level	Limit of detection	Signal theshold
Polarity mode	Negative	RT verified by standard	Yes
Type of negative (precursor)ion	[M-H]-	Separation of isobaric/isomeric interferece confirmed	No
Fragments for identification		Model for separation prediction	No

Fragment name
FA1(-H)
FA2(-H)
GP(153)
P(79)

Isotope correction at MS1	No	Additional dimension/techniques	-
Isotope correction at MS2	No	Lipid Identification Software	manually
MS1 verified by standard	Yes	Data manipulation	-
MS2 verified by standard	Yes	Nomenclature for intact lipid molecule	Yes
Background check at MS1	Yes	Nomenclature for fragment ions	N/A
Background check at MS2	Yes	Further identification remarks	Proposed identifications were further validated by plotting the retention time of lipid species against their Kendrick mass defect by hydrogen and lipid species not following expected trendlines were excluded.
Did you presume assumptions for identification?	Yes		

7) PA[M-H]- / Lipid quantification

Quantitative	No	Batch correction	No
Normalization to reference	Yes	Further quantification remarks	Individual lipid species concentrations were normalized to the wet tissue weight.

8) PS[M-H]- / Lipid identification

Lipid class	PS	Did you presume assumptions for identification?	No
MS Level for identification	MS1, MS2	Check on:	Isomeric overlap, Isobaric overlap, In-source fragmentation
Identification level	Molecular species level	Limit of detection	Signal theshold
Polarity mode	Negative	RT verified by standard	Yes
Type of negative (precursor)ion	[M-H]-	Separation of isobaric/isomeric interferece confirmed	No
Fragments for identification		Model for separation prediction	No
Isotope correction at MS1	No	Additional dimension/techniques	-
Isotope correction at MS2	Type 2	Lipid Identification Software	LipidHunter
MS1 verified by standard	Yes	Data manipulation	-
MS2 verified by standard	Yes	Nomenclature for intact lipid molecule	Yes
Background check at MS1	Yes	Nomenclature for fragment ions	N/A
Background check at MS2	Yes	Further identification remarks	Proposed identifications were further validated by plotting the retention time of lipid species against their Kendrick mass defect by hydrogen and lipid species not following expected trendlines were excluded.

Fragment name
-(C3H5NO2,87)
FA1(-H)
FA2(-H)
GP(153)

8) PS[M-H]- / Lipid quantification

Quantitative	No	Batch correction	No
Normalization to reference	Yes	Further quantification remarks	Individual lipid species concentrations were normalized to the wet tissue weight.

9) PI[M-H]- / Lipid identification

Lipid class	PI	Did you presume assumptions for identification?	No
MS Level for identification	MS1, MS2	Check on:	Isomeric overlap, Isobaric overlap, In-source fragmentation
Identification level	Molecular species level	Limit of detection	Signal theshold
Polarity mode	Negative	RT verified by standard	Yes
Type of negative (precursor)ion	[M-H]-	Separation of isobaric/isomeric interferece confirmed	No
Fragments for identification		Model for separation prediction	No

Fragment name
FA1(-H)
FA2(-H)
HG(PI,241)-(H2O)
-FA1(-H)
-FA2(-H)

Isotope correction at MS1	No	Additional dimension/techniques	-
Isotope correction at MS2	Type 2	Lipid Identification Software	LipidHunter
MS1 verified by standard	Yes	Data manipulation	-
MS2 verified by standard	Yes	Nomenclature for intact lipid molecule	Yes
Background check at MS1	Yes	Nomenclature for fragment ions	N/A
Background check at MS2	Yes	Further identification remarks	Proposed identifications were further validated by plotting the retention time of lipid species against their Kendrick mass defect by hydrogen and lipid species not following expected trendlines were excluded.

9) PI[M-H]- / Lipid quantification

Quantitative	No	Batch correction	No
Normalization to reference	Yes	Further quantification remarks	Individual lipid species concentrations were normalized to the wet tissue weight.

10) PG[M-H]- / Lipid identification

Lipid class	PG	Did you presume assumptions for identification?	No
MS Level for identification	MS1, MS2	Check on:	Isomeric overlap, Isobaric overlap, In-source fragmentation
Identification level	Molecular species level	Limit of detection	Signal theshold
Polarity mode	Negative	RT verified by standard	Yes
Type of negative (precursor)ion	[M-H]-	Separation of isobaric/isomeric interferece confirmed	No
Fragments for identification	Fragment name: FA1(-H), FA2(-H), GP(153), -FA1(+HO), -FA2(+HO)	Model for separation prediction	No
Isotope correction at MS1	No	Additional dimension/techniques	-
Isotope correction at MS2	Type 2	Lipid Identification Software	LipidHunter
MS1 verified by standard	Yes	Data manipulation	-
MS2 verified by standard	Yes	Nomenclature for intact lipid molecule	Yes
Background check at MS1	Yes	Nomenclature for fragment ions	N/A
Background check at MS2	Yes	Further identification remarks	Proposed identifications were further validated by plotting the retention time of lipid species against their Kendrick mass defect by hydrogen and lipid species not following expected trendlines were excluded.

10) PG[M-H]- / Lipid quantification

Quantitative	No	Batch correction	No
Normalization to reference	Yes	Further quantification remarks	Individual lipid species concentrations were normalized to the wet tissue weight.

11) CL[M-H]- / Lipid identification

Lipid class	CL	Did you presume assumptions for identification?	No
MS Level for identification	MS1, MS2	Check on:	Isomeric overlap, Isobaric overlap, In-source fragmentation
Identification level	Molecular species level	Limit of detection	Signal theshold
Polarity mode	Negative	RT verified by standard	Yes
Type of negative (precursor)ion	[M-H]-	Separation of isobaric/isomeric interferece confirmed	No
Fragments for identification		Model for separation prediction	No

Fragment name
-PG
-PG-FA1
-PG-FA1-(H2O)
FA1(-H)
FA2(-H)
GP(153)

Isotope correction at MS1	No	Additional dimension/techniques	-
Isotope correction at MS2	Type 2	Lipid Identification Software	manually
MS1 verified by standard	Yes	Data manipulation	-
MS2 verified by standard	Yes	Nomenclature for intact lipid molecule	Yes
Background check at MS1	Yes	Nomenclature for fragment ions	N/A
Background check at MS2	Yes	Further identification remarks	Proposed identifications were further validated by plotting the retention time of lipid species against their Kendrick mass defect by hydrogen and lipid species not following expected trendlines were excluded.

11) CL[M-H]- / Lipid quantification

Quantitative	No	Batch correction	No
Normalization to reference	Yes	Further quantification remarks	Individual lipid species concentrations were normalized to the wet tissue weight.

12) LPC[M+HCOO]- / Lipid identification

Lipid class	LPC	Did you presume assumptions for identification?	No
MS Level for identification	MS1, MS2	Check on:	Isomeric overlap, Isobaric overlap, In-source fragmentation
Identification level	Molecular species level	Limit of detection	Signal theshold
Polarity mode	Negative	RT verified by standard	Yes
Type of negative (precursor)ion	[M+HCOO]-	Separation of isobaric/isomeric interferece confirmed	No
Fragments for identification		Model for separation prediction	No

Fragment name
-(CH3+HCOO)
FA1(-H)
HG(PC,224)

Isotope correction at MS1	No	Additional dimension/techniques	-
Isotope correction at MS2	No	Lipid Identification Software	LipidHunter
MS1 verified by standard	Yes	Data manipulation	-
MS2 verified by standard	Yes	Nomenclature for intact lipid molecule	Yes
Background check at MS1	Yes	Nomenclature for fragment ions	N/A
Background check at MS2	Yes	Further identification remarks	Proposed identifications were further validated by plotting the retention time of lipid species against their Kendrick mass defect by hydrogen and lipid species not following expected trendlines were excluded.

12) LPC[M+HCOO]- / Lipid quantification

Quantitative	Yes	Limit of quantification	Linear regression analysis was applied by plotting the calculated concentrations against their AUC value to identify and exclude possible outliers and features showing non-linear behaviour.
MS Level for quantification	MS1	Normalization to reference	Yes
Internal lipid standard(s) MS1		Lipid Quantification Software	Skyline

Internal standard	Endogenous subclass
LPC 18:1 (d7)	LPC

Type of quantification	Internal standard amount	Batch correction	No
Response correction	No	Further quantification remarks	Individual lipid species concentrations were normalized to the wet tissue weight.
Type I isotope correction	Yes		

13) LPE[M-H]- / Lipid identification

Lipid class	LPE	Did you presume assumptions for identification?	No
MS Level for identification	MS1, MS2	Check on:	Isomeric overlap, Isobaric overlap, In-source fragmentation
Identification level	Molecular species level	Limit of detection	Signal theshold
Polarity mode	Negative	RT verified by standard	Yes
Type of negative (precursor)ion	[M-H]-	Separation of isobaric/isomeric interferece confirmed	No
Fragments for identification		Model for separation prediction	No

Fragment name
FA1(-H)
HG(PE,196)
HG(PE,140)

Isotope correction at MS1	No	Additional dimension/techniques	-
Isotope correction at MS2	No	Lipid Identification Software	LipidHunter
MS1 verified by standard	Yes	Data manipulation	-
MS2 verified by standard	Yes	Nomenclature for intact lipid molecule	Yes
Background check at MS1	Yes	Nomenclature for fragment ions	N/A
Background check at MS2	Yes	Further identification remarks	Proposed identifications were further validated by plotting the retention time of lipid species against their Kendrick mass defect by hydrogen and lipid species not following expected trendlines were excluded.

13) LPE[M-H]- / Lipid quantification

Quantitative	Yes	Limit of quantification	Linear regression analysis was applied by plotting the calculated concentrations against their AUC value to identify and exclude possible outliers and features showing non-linear behaviour.
MS Level for quantification	MS1	Normalization to reference	Yes
Internal lipid standard(s) MS1		Lipid Quantification Software	Skyline

Internal standard	Endogenous subclass
LPE 18:1 (d7)	LPE

Type of quantification	Internal standard amount	Batch correction	No
Response correction	No	Further quantification remarks	Individual lipid species concentrations were normalized to the wet tissue weight.
Type I isotope correction	Yes		

14) LPA[M-H]- / Lipid identification

Lipid class	LPA	Did you presume assumptions for identification?	No
MS Level for identification	MS1, MS2	Check on:	Isomeric overlap, Isobaric overlap, In-source fragmentation
Identification level	Molecular species level	Limit of detection	Signal theshold
Polarity mode	Negative	RT verified by standard	Yes
Type of negative (precursor)ion	[M-H]-	Separation of isobaric/isomeric interferece confirmed	No
Fragments for identification		Model for separation prediction	No

Fragment name
FA1(-H)
GP(153)
P(79)

Isotope correction at MS1	No	Additional dimension/techniques	-
Isotope correction at MS2	No	Lipid Identification Software	manually
MS1 verified by standard	Yes	Data manipulation	-
MS2 verified by standard	Yes	Nomenclature for intact lipid molecule	Yes
Background check at MS1	Yes	Nomenclature for fragment ions	N/A
Background check at MS2	Yes	Further identification remarks	Proposed identifications were further validated by plotting the retention time of lipid species against their Kendrick mass defect by hydrogen and lipid species not following expected trendlines were excluded.

14) LPA[M-H]- / Lipid quantification

Quantitative	No	Batch correction	No
Normalization to reference	Yes	Further quantification remarks	Individual lipid species concentrations were normalized to the wet tissue weight.

15) LPI[M-H]- / Lipid identification

Lipid class	LPI	Did you presume assumptions for identification?	No
MS Level for identification	MS1, MS2	Check on:	Isomeric overlap, Isobaric overlap, In-source fragmentation
Identification level	Molecular species level	Limit of detection	Signal theshold
Polarity mode	Negative	RT verified by standard	Yes
Type of negative (precursor)ion	[M-H]-	Separation of isobaric/isomeric interferece confirmed	No
Fragments for identification		Model for separation prediction	No

Fragment name
FA(-H)
HG(PI,241)
GP(153)

Isotope correction at MS1	No	Additional dimension/techniques	-
Isotope correction at MS2	No	Lipid Identification Software	LipidHunter
MS1 verified by standard	No	Data manipulation	-
MS2 verified by standard	No	Nomenclature for intact lipid molecule	Yes
Background check at MS1	Yes	Nomenclature for fragment ions	N/A
Background check at MS2	Yes	Further identification remarks	Proposed identifications were further validated by plotting the retention time of lipid species against their Kendrick mass defect by hydrogen and lipid species not following expected trendlines were excluded.

15) LPI[M-H]- / Lipid quantification

Quantitative	No	Batch correction	No
Normalization to reference	Yes	Further quantification remarks	Individual lipid species concentrations were normalized to the wet tissue weight.

16) LPS[M-H]- / Lipid identification

Lipid class	LPS	Did you presume assumptions for identification?	No
MS Level for identification	MS1, MS2	Check on:	Isomeric overlap, Isobaric overlap, In-source fragmentation
Identification level	Molecular species level	Limit of detection	Signal theshold
Polarity mode	Negative	RT verified by standard	Yes
Type of negative (precursor)ion	[M-H]-	Separation of isobaric/isomeric interferece confirmed	No
Fragments for identification		Model for separation prediction	No

Fragment name
FA1(-H)
-(C3H5NO2,87)
GP(153)

Isotope correction at MS1	No	Additional dimension/techniques	-
Isotope correction at MS2	No	Lipid Identification Software	LipidHunter
MS1 verified by standard	No	Data manipulation	-
MS2 verified by standard	No	Nomenclature for intact lipid molecule	Yes
Background check at MS1	Yes	Nomenclature for fragment ions	N/A
Background check at MS2	Yes	Further identification remarks	Proposed identifications were further validated by plotting the retention time of lipid species against their Kendrick mass defect by hydrogen and lipid species not following expected trendlines were excluded.

16) LPS[M-H]- / Lipid quantification

Quantitative	No	Batch correction	No
Normalization to reference	Yes	Further quantification remarks	Individual lipid species concentrations were normalized to the wet tissue weight.

17) SM[M+H]+ / Lipid identification

Lipid class	SM	Did you presume assumptions for identification?	No
MS Level for identification	MS1, MS2	Check on:	Isomeric overlap, Isobaric overlap, In-source fragmentation
Identification level	Species level	Limit of detection	Signal theshold
Polarity mode	Positive	RT verified by standard	Yes
Type of positive (precursor)ion	[M+H]+	Separation of isobaric/isomeric interferece confirmed	No
Fragments for identification	Fragment name: -(H2O,18); LCB(-H3O2); HG(PC,184)	Model for separation prediction	No
Isotope correction at MS1	No	Additional dimension/techniques	-
Isotope correction at MS2	No	Lipid Identification Software	LipidHunter
MS1 verified by standard	Yes	Data manipulation	-
MS2 verified by standard	Yes	Nomenclature for intact lipid molecule	Yes
Background check at MS1	Yes	Nomenclature for fragment ions	N/A
Background check at MS2	Yes	Further identification remarks	Proposed identifications were further validated by plotting the retention time of lipid species against their Kendrick mass defect by hydrogen and lipid species not following expected trendlines were excluded.

17) SM[M+H]+ / Lipid quantification

Quantitative	Yes	Limit of quantification	Linear regression analysis was applied by plotting the calculated concentrations against their AUC value to identify and exclude possible outliers and features showing non-linear behaviour.
MS Level for quantification	MS1	Normalization to reference	Yes
Internal lipid standard(s) MS1	Internal standard: SM 18:1;O2/18:1 (d9) — Endogenous subclass: SM	Lipid Quantification Software	Skyline
Type of quantification	Internal standard amount	Batch correction	No
Response correction	No	Further quantification remarks	Individual lipid species concentrations were normalized to the wet tissue weight.
Type I isotope correction	Yes		

18) Cer[M+H]+ / Lipid identification

Lipid class	Cer	Did you presume assumptions for identification?	No
MS Level for identification	MS1, MS2	Check on:	Isomeric overlap, Isobaric overlap, In-source fragmentation
Identification level	Molecular species level	Limit of detection	Signal theshold
Polarity mode	Positive	RT verified by standard	Yes
Type of positive (precursor)ion	[M+H]+	Separation of isobaric/isomeric interferece confirmed	No
Fragments for identification		Model for separation prediction	No
Isotope correction at MS1	No	Additional dimension/techniques	-
Isotope correction at MS2	No	Lipid Identification Software	LipidHunter
MS1 verified by standard	Yes	Data manipulation	-
MS2 verified by standard	Yes	Nomenclature for intact lipid molecule	Yes
Background check at MS1	Yes	Nomenclature for fragment ions	N/A
Background check at MS2	Yes	Further identification remarks	Proposed identifications were further validated by plotting the retention time of lipid species against their Kendrick mass defect by hydrogen and lipid species not following expected trendlines were excluded.

Fragments for identification:

Fragment name
-(H2O,18)
LCB(-HO)
LCB(-H3O2)
LCB(+H)
FA(-OH+NH3)

18) Cer[M+H]+ / Lipid quantification

Quantitative	Yes	Limit of quantification	Linear regression analysis was applied by plotting the calculated concentrations against their AUC value to identify and exclude possible outliers and features showing non-linear behaviour.
MS Level for quantification	MS1	Normalization to reference	Yes
Internal lipid standard(s) MS1		Lipid Quantification Software	Skyline
Type of quantification	Internal standard amount	Batch correction	No
Response correction	No	Further quantification remarks	Individual lipid species concentrations were normalized to the wet tissue weight.
Type I isotope correction	Yes		

Internal lipid standard(s) MS1:

Internal standard	Endogenous subclass
Cer 18:1;O2/12:0	Cer
Cer 18:1;O2/25:0	Cer
Cer 18:1;O/17:0;O	Cer
Cer 18:0;O3/8:0	Cer
Cer 18:0;O2/12:0	Cer
Cer 18:0;O2/8:0	Cer

19) HexCer[M+H]+ / Lipid identification

Lipid class	HexCer	Did you presume assumptions for identification?	No
MS Level for identification	MS1, MS2	Check on:	Isomeric overlap, Isobaric overlap, In-source fragmentation
Identification level	Molecular species level	Limit of detection	Signal theshold
Polarity mode	Positive	RT verified by standard	Yes
Type of positive (precursor)ion	[M+H]+	Separation of isobaric/isomeric interferece confirmed	No
Fragments for identification		Model for separation prediction	No

Fragment name
-(H2O,18)
-HG(Hex,180)
-HG(Hex,198)
-HG(Hex,162)
LCB(-HO)
LCB(-H3O2)
LCB(-CH3O2)
FA(-OH+NH3)

Isotope correction at MS1	No	Additional dimension/techniques	-
Isotope correction at MS2	No	Lipid Identification Software	LipidHunter
MS1 verified by standard	Yes	Data manipulation	-
MS2 verified by standard	Yes	Nomenclature for intact lipid molecule	Yes
Background check at MS1	Yes	Nomenclature for fragment ions	N/A
Background check at MS2	Yes	Further identification remarks	Proposed identifications were further validated by plotting the retention time of lipid species against their Kendrick mass defect by hydrogen and lipid species not following expected trendlines were excluded.

19) HexCer[M+H]+ / Lipid quantification

Quantitative	Yes	Limit of quantification	Linear regression analysis was applied by plotting the calculated concentrations against their AUC value to identify and exclude possible outliers and features showing non-linear behaviour.
MS Level for quantification	MS1	Normalization to reference	Yes
Internal lipid standard(s) MS1		Lipid Quantification Software	Skyline
Type of quantification	Internal standard amount	Batch correction	No
Response correction	No	Further quantification remarks	Individual lipid species concentrations were normalized to the wet tissue weight.
Type I isotope correction	Yes		

Internal lipid standard(s) MS1:

Internal standard	Endogenous subclass
GlcCer d18:1;O2/12:0	HexCer

20) Hex2Cer[M+H]+ / Lipid identification

Lipid class	Hex2Cer	Did you presume assumptions for identification?	No
MS Level for identification	MS1, MS2	Check on:	Isomeric overlap, Isobaric overlap, In-source fragmentation
Identification level	Molecular species level	Limit of detection	Signal theshold
Polarity mode	Positive	RT verified by standard	Yes
Type of positive (precursor)ion	[M+H]+	Separation of isobaric/isomeric interferece confirmed	No
Fragments for identification		Model for separation prediction	No

Fragment name
-(H2O,18)
-HG(Hex2,342)
-HG(Hex2,324)
-HG(Hex2,360)
LCB(-HO)
LCB(-H3O2)
LCB(-CH3O2)
FA(-OH+NH3)

Isotope correction at MS1	No	Additional dimension/techniques	-
Isotope correction at MS2	No	Lipid Identification Software	LipidHunter
MS1 verified by standard	Yes	Data manipulation	-
MS2 verified by standard	Yes	Nomenclature for intact lipid molecule	Yes
Background check at MS1	Yes	Nomenclature for fragment ions	N/A
Background check at MS2	Yes	Further identification remarks	Proposed identifications were further validated by plotting the retention time of lipid species against their Kendrick mass defect by hydrogen and lipid species not following expected trendlines were excluded.

20) Hex2Cer[M+H]+ / Lipid quantification

Quantitative	Yes	Limit of quantification	Linear regression analysis was applied by plotting the calculated concentrations against their AUC value to identify and exclude possible outliers and features showing non-linear behaviour.
MS Level for quantification	MS1	Normalization to reference	Yes
Internal lipid standard(s) MS1		Lipid Quantification Software	Skyline
Type of quantification	Internal standard amount	Batch correction	No
Response correction	No	Further quantification remarks	Individual lipid species concentrations were normalized to the wet tissue weight.
Type I isotope correction	Yes		

Internal standard	Endogenous subclass
LacCer 18:1;O2/12:0	Hex2Cer

21) Hex3Cer[M+H]+ / Lipid identification

Lipid class	Hex3Cer	Did you presume assumptions for identification?	No
MS Level for identification	MS1, MS2	Check on:	Isomeric overlap, Isobaric overlap, In-source fragmentation
Identification level	Molecular species level	Limit of detection	Signal theshold
Polarity mode	Positive	RT verified by standard	Yes
Type of positive (precursor)ion	[M+H]+	Separation of isobaric/isomeric interferece confirmed	No
Fragments for identification		Model for separation prediction	No
Isotope correction at MS1	No	Additional dimension/techniques	-
Isotope correction at MS2	No	Lipid Identification Software	LipidHunter
MS1 verified by standard	No	Data manipulation	-
MS2 verified by standard	No	Nomenclature for intact lipid molecule	Yes
Background check at MS1	Yes	Nomenclature for fragment ions	N/A
Background check at MS2	Yes	Further identification remarks	Proposed identifications were further validated by plotting the retention time of lipid species against their Kendrick mass defect by hydrogen and lipid species not following expected trendlines were excluded.

Fragment name
-(H2O,18)
-HG(Hex,180)
-HG(Hex3,504)
-HG(Hex3,522)
-HG(Hex3,486)
LCB(-HO)
LCB(-H3O2)

21) Hex3Cer[M+H]+ / Lipid quantification

Quantitative	Yes	Limit of quantification	Linear regression analysis was applied by plotting the calculated concentrations against their AUC value to identify and exclude possible outliers and features showing non-linear behaviour.
MS Level for quantification	MS1	Normalization to reference	Yes
Internal lipid standard(s) MS1		Lipid Quantification Software	Skyline
Type of quantification	Internal standard amount	Batch correction	No
Response correction	No	Further quantification remarks	Individual lipid species concentrations were normalized to the wet tissue weight.
Type I isotope correction	Yes		

Internal standard	Endogenous subclass
LacCer 18:1;O2/12:0	Hex3Cer

22) GM3[M-H]- / Lipid identification

Lipid class	GM3	Did you presume assumptions for identification?	No
MS Level for identification	MS1, MS2	Check on:	Isomeric overlap, Isobaric overlap, In-source fragmentation
Identification level	Species level	Limit of detection	Signal theshold
Polarity mode	Negative	RT verified by standard	Yes
Type of negative (precursor)ion	[M-H]-	Separation of isobaric/isomeric interferece confirmed	No
Fragments for identification		Model for separation prediction	No
Isotope correction at MS1	No	Additional dimension/techniques	-
Isotope correction at MS2	Type 2	Lipid Identification Software	manually
MS1 verified by standard	Yes	Data manipulation	-
MS2 verified by standard	Yes	Nomenclature for intact lipid molecule	Yes
Background check at MS1	Yes	Nomenclature for fragment ions	N/A
Background check at MS2	Yes	Further identification remarks	Proposed identifications were further validated by plotting the retention time of lipid species against their Kendrick mass defect by hydrogen and lipid species not following expected trendlines were excluded.

Fragment name
-HG(NHex,291)
HG(NHex,290)
-HG(NHex3,615)

22) GM3[M-H]- / Lipid quantification

Quantitative	No	Batch correction	No
Normalization to reference	Yes	Further quantification remarks	Individual lipid species concentrations were normalized to the wet tissue weight.

Supplementary Note 2 | Lipidomics Minimal Reporting Checklist with details on the AV lipid quantification using Folch extraction method and targeted lipidomics workflow.

Contents of Report

Created by https://lipidomicstandards.org, version v2.4.0

Separation Workflow

Overall study design

Title of the study	Sex-specific lipidomic signatures in aortic valve disease reflect differential fibro-calcific progression (targeted, Folch)		
Document creation date	12/20/2024	Corresponding Email	maria.fedorova@tu-dresden.de
Principal investigator	Maria Fedorova	Is the workflow targeted or untargeted?	Targeted
Institution	Technical University Dresden, Germany	Clinical	No

Lipid extraction

Extraction method	2-phase system	Were internal standards added prior extraction?	Yes
pH adjustment	None	Special conditions	All solvents used for extraction were supplemented with 0.1 % (w/v) BHT. All extraction steps were performed on ice.
2-phase system	Folch	Derivatization	-

Analytical platform

Ionizaton additives	Ammonium formate, Formic acid	Mass resolution for detected ion at MS1	Low resolution
Number of separation dimensions	One dimension	Resolution at MS1	Unit
Separation type 1	LC	Recording mode of raw data at MS1	Profile mode
Separation mode 1 (liquid)	RP	Mass window for precursor ion isolation (in Da total isolation window)	0.7
Detector	Mass spectrometer	Mass resolution for detected ion at MS2	Low resolution
MS type	QQQ	Resolution at MS2	Unit
MS vendor	Thermo	Recording mode of raw data at MS2	Profile mode
Ion source	ESI	Was/Were additional dimension/techniques used	No
MS Level	MS1, MS2		

Quality control

Blanks	Yes	Quality control	Yes
Type of Blanks	Solvent blank, Internal standard blank	Type of QC sample	Sample pool

Method qualification and validation

Method validation	Yes	Precision	No
Lipid recovery	Yes	Accuracy	No
Dynamic quantification range	Yes	Guidelines followed	None
Limit of quantitation (LOQ)/Limit of detection (LOD)	Yes		

Reporting

Are reported raw data uploaded into repository?	Yes	Summary data	Quantification and identification data
Link to repository / ID to entry	doi: 10.21228/M8G24F	Raw data upload	Yes
Are metadata available?	Yes	Additional comments	-

Sample Descriptions

Aortic valve tissue / Human / Tissues (e.g., liver, heart, brain)

Perfusion	No	Additives	None
Storage and collection conditions	Available	Were samples stored under inert gas?	No
Provided preanalytical information	Freeze-thaw cycles	Additional preservation methods	No
Temperature handling original sample	Room temperature	Biobank samples	No
Instant sample preparation	No	Sample homogenization	Yes
Storage temperature	-80 °C	Sample homogenization solvent	liquid nitrogen
Freeze-thaw cycles	1		

Lipid Class Descriptions

1) PA[M-H]- / Lipid identification

Lipid class	PA	Which assumptions were presumed?	It was checked that the identified PA is of endogeneous origin and not the result of in-source fragmentation of other PL.
MS Level for identification	MS1, MS2	Check on:	Isomeric overlap, Isobaric overlap, In-source fragmentation
Identification level	Molecular species level	Limit of detection	Signal theshold
Polarity mode	Negative	RT verified by standard	Yes
Type of negative (precursor)ion	[M-H]-	Separation of isobaric/isomeric interferece confirmed	No
Fragments for identification	Fragment name: FA1(-H), FA2(-H), GP(153), P(79)	Model for separation prediction	No
Isotope correction at MS1	No	Additional dimension/techniques	-
Isotope correction at MS2	No	Lipid Identification Software	manually
MS1 verified by standard	Yes	Data manipulation	-
MS2 verified by standard	Yes	Nomenclature for intact lipid molecule	Yes
Background check at MS1	Yes	Nomenclature for fragment ions	N/A
Background check at MS2	Yes	Further identification remarks	Proposed identifications were further validated by plotting the retention time of lipid species against their Kendrick mass defect by hydrogen and lipid species not following expected trendlines were excluded.
Did you presume assumptions for identification?	Yes		

1) PA[M-H]- / Lipid quantification

Quantitative	Yes	Limit of quantification	Linear regression analysis was applied by plotting the calculated concentrations against their AUC value to identify and exclude possible outliers and features showing non-linear behaviour.
MS Level for quantification	MS2	Normalization to reference	Yes
Internal lipid standard(s) MS2		Lipid Quantification Software	Skyline
Type of quantification	Internal standard amount	Batch correction	No
Response correction	No	Further quantification remarks	Individual lipid species concentrations were normalized to the wet tissue weight.
Type I isotope correction	Yes		

Internal lipid standard(s) MS2:

Internal standard	Fragment(s)	Endogenous subclass
PA 15:0/18:1 (d7)	FA1(-H)	PA
PA 15:0/18:1 (d7)	FA2(-H)	PA
PA 15:0/18:1 (d7)	GP(153)	PA

2) PS[M-H]- / Lipid identification

Lipid class	PS	Did you presume assumptions for identification?	No
MS Level for identification	MS1, MS2	Check on:	Isomeric overlap, Isobaric overlap, In-source fragmentation
Identification level	Molecular species level	Limit of detection	Signal theshold
Polarity mode	Negative	RT verified by standard	Yes
Type of negative (precursor)ion	[M-H]-	Separation of isobaric/isomeric interferece confirmed	No
Fragments for identification		Model for separation prediction	No
Isotope correction at MS1	No	Additional dimension/techniques	-
Isotope correction at MS2	Type 2	Lipid Identification Software	LipidHunter
MS1 verified by standard	Yes	Data manipulation	-
MS2 verified by standard	Yes	Nomenclature for intact lipid molecule	Yes
Background check at MS1	Yes	Nomenclature for fragment ions	N/A
Background check at MS2	Yes	Further identification remarks	Proposed identifications were further validated by plotting the retention time of lipid species against their Kendrick mass defect by hydrogen and lipid species not following expected trendlines were excluded.

Fragments for identification:

Fragment name
-(C3H5NO2,87)
FA1(-H)
FA2(-H)
GP(153)

2) PS[M-H]- / Lipid quantification

Quantitative	Yes	Limit of quantification	Linear regression analysis was applied by plotting the calculated concentrations against their AUC value to identify and exclude possible outliers and features showing non-linear behaviour.
MS Level for quantification	MS2	Normalization to reference	Yes
Internal lipid standard(s) MS2		Lipid Quantification Software	Skyline
Type of quantification	Internal standard amount	Batch correction	No
Response correction	No	Further quantification remarks	Individual lipid species concentrations were normalized to the wet tissue weight.
Type I isotope correction	Yes		

Internal lipid standard(s) MS2:

Internal standard	Fragment(s)	Endogenous subclass
PS 15:0/18:1 (d7)	-(C3H5NO2,87)	PS
PS 15:0/18:1 (d7)	FA1(-H)	PS
PS 15:0/18:1 (d7)	FA2(-H)	PS
PS 15:0/18:1 (d7)	GP(153)	PS

3) PI[M-H]- / Lipid identification

Lipid class	PI	Did you presume assumptions for identification?	No
MS Level for identification	MS1, MS2	Check on:	Isomeric overlap, Isobaric overlap, In-source fragmentation
Identification level	Molecular species level	Limit of detection	Signal theshold
Polarity mode	Negative	RT verified by standard	Yes
Type of negative (precursor)ion	[M-H]-	Separation of isobaric/isomeric interferece confirmed	No
Fragments for identification	**Fragment name**<br>FA1(-H)<br>FA2(-H)<br>HG(PI,241)-(H2O)<br>-FA1(-H)<br>-FA2(-H)	Model for separation prediction	No
Isotope correction at MS1	No	Additional dimension/techniques	-
Isotope correction at MS2	Type 2	Lipid Identification Software	LipidHunter
MS1 verified by standard	Yes	Data manipulation	-
MS2 verified by standard	Yes	Nomenclature for intact lipid molecule	Yes
Background check at MS1	Yes	Nomenclature for fragment ions	N/A
Background check at MS2	Yes	Further identification remarks	Proposed identifications were further validated by plotting the retention time of lipid species against their Kendrick mass defect by hydrogen and lipid species not following expected trendlines were excluded.

3) PI[M-H]- / Lipid quantification

Quantitative	Yes	Limit of quantification	Linear regression analysis was applied by plotting the calculated concentrations against their AUC value to identify and exclude possible outliers and features showing non-linear behaviour.
MS Level for quantification	MS2	Normalization to reference	Yes
Internal lipid standard(s) MS2		Lipid Quantification Software	Skyline

Internal standard	Fragment(s)	Endogenous subclass
PI 15:0/18:1 (d7)	FA1(-H)	PI
PI 15:0/18:1 (d7)	FA2(-H)	PI
PI 15:0/18:1 (d7)	HG(PI,241)-(H2O)	

Type of quantification	Internal standard amount	Batch correction	No
Response correction	No	Further quantification remarks	Individual lipid species concentrations were normalized to the wet tissue weight.
Type I isotope correction	Yes		

4) PG[M-H]- / Lipid identification

Lipid class	PG	Did you presume assumptions for identification?	No
MS Level for identification	MS1, MS2	Check on:	Isomeric overlap, Isobaric overlap, In-source fragmentation
Identification level	Molecular species level	Limit of detection	Signal theshold
Polarity mode	Negative	RT verified by standard	Yes
Type of negative (precursor)ion	[M-H]-	Separation of isobaric/isomeric interferece confirmed	No
Fragments for identification		Model for separation prediction	No

Fragment name
FA1(-H)
FA2(-H)
GP(153)
-FA1(+HO)
-FA2(+HO)

Isotope correction at MS1	No	Additional dimension/techniques	-
Isotope correction at MS2	Type 2	Lipid Identification Software	LipidHunter
MS1 verified by standard	Yes	Data manipulation	-
MS2 verified by standard	Yes	Nomenclature for intact lipid molecule	Yes
Background check at MS1	Yes	Nomenclature for fragment ions	N/A
Background check at MS2	Yes	Further identification remarks	Proposed identifications were further validated by plotting the retention time of lipid species against their Kendrick mass defect by hydrogen and lipid species not following expected trendlines were excluded.

4) PG[M-H]- / Lipid quantification

Quantitative	Yes	Limit of quantification	Linear regression analysis was applied by plotting the calculated concentrations against their AUC value to identify and exclude possible outliers and features showing non-linear behaviour.
MS Level for quantification	MS2	Normalization to reference	Yes
Internal lipid standard(s) MS2		Lipid Quantification Software	Skyline
Type of quantification	Internal standard amount	Batch correction	No
Response correction	No	Further quantification remarks	Individual lipid species concentrations were normalized to the wet tissue weight.
Type I isotope correction	Yes		

Internal lipid standard(s) MS2:

Internal standard	Fragment(s)	Endogenous subclass
PG 15:0/18:1 (d7)	FA1(-H)	PG
PG 15:0/18:1 (d7)	FA2(-H)	PG
PG 15:0/18:1 (d7)	GP(153)	PG

5) CL[M-H]- / Lipid identification

Lipid class	CL	Did you presume assumptions for identification?	No
MS Level for identification	MS1, MS2	Check on:	Isomeric overlap, Isobaric overlap, In-source fragmentation
Identification level	Molecular species level	Limit of detection	Signal theshold
Polarity mode	Negative	RT verified by standard	Yes
Type of negative (precursor)ion	[M-H]-	Separation of isobaric/isomeric interferece confirmed	No
Fragments for identification		Model for separation prediction	No

Fragment name
-PG
-PG-FA1
-PG-FA1-(H2O)
FA1(-H)
FA2(-H)
GP(153)

Isotope correction at MS1	No	Additional dimension/techniques	-
Isotope correction at MS2	Type 2	Lipid Identification Software	manually
MS1 verified by standard	Yes	Data manipulation	-
MS2 verified by standard	Yes	Nomenclature for intact lipid molecule	Yes
Background check at MS1	Yes	Nomenclature for fragment ions	N/A
Background check at MS2	Yes	Further identification remarks	Proposed identifications were further validated by plotting the retention time of lipid species against their Kendrick mass defect by hydrogen and lipid species not following expected trendlines were excluded.

5) CL[M-H]- / Lipid quantification

Quantitative	Yes	Limit of quantification	Linear regression analysis was applied by plotting the calculated concentrations against their AUC value to identify and exclude possible outliers and features showing non-linear behaviour.
MS Level for quantification	MS2	Normalization to reference	Yes
Internal lipid standard(s) MS2		Lipid Quantification Software	Skyline
Type of quantification	Internal standard amount	Batch correction	No
Response correction	No	Further quantification remarks	Individual lipid species concentrations were normalized to the wet tissue weight.
Type I isotope correction	Yes		

Internal lipid standard(s) MS2:

Internal standard	Fragment(s)	Endogenous subclass
CL 18:2/18:2/18:2/18:2 (d5)	FA1(-H)	CL
CL 18:2/18:2/18:2/18:2 (d5)	FA2(-H)	CL
CL 18:2/18:2/18:2/18:2 (d5)	GP(153)	CL

6) LPA[M-H]- / Lipid identification

Lipid class	LPA	Did you presume assumptions for identification?	No
MS Level for identification	MS1, MS2	Check on:	Isomeric overlap, Isobaric overlap, In-source fragmentation
Identification level	Molecular species level	Limit of detection	Signal theshold
Polarity mode	Negative	RT verified by standard	Yes
Type of negative (precursor)ion	[M-H]-	Separation of isobaric/isomeric interferece confirmed	No
Fragments for identification		Model for separation prediction	No

Fragment name
FA1(-H)
GP(153)
P(79)

Isotope correction at MS1	No	Additional dimension/techniques	-
Isotope correction at MS2	No	Lipid Identification Software	manually
MS1 verified by standard	Yes	Data manipulation	-
MS2 verified by standard	Yes	Nomenclature for intact lipid molecule	Yes
Background check at MS1	Yes	Nomenclature for fragment ions	N/A
Background check at MS2	Yes	Further identification remarks	Proposed identifications were further validated by plotting the retention time of lipid species against their Kendrick mass defect by hydrogen and lipid species not following expected trendlines were excluded.

6) LPA[M-H]- / Lipid quantification

Quantitative	Yes	Limit of quantification	Linear regression analysis was applied by plotting the calculated concentrations against their AUC value to identify and exclude possible outliers and features showing non-linear behaviour.
MS Level for quantification	MS2	Normalization to reference	Yes
Internal lipid standard(s) MS2		Lipid Quantification Software	Skyline

Internal standard	Fragment(s)	Endogenous subclass
LPA 17:1	FA1(-H)	LPA
LPA 17:1	GP(153)	LPA
LPA 17:1	P(79)	LPA

Type of quantification	Internal standard amount	Batch correction	No
Response correction	No	Further quantification remarks	Individual lipid species concentrations were normalized to the wet tissue weight.
Type I isotope correction	Yes		

7) LPI[M-H]- / Lipid identification

Lipid class	LPI	Did you presume assumptions for identification?	No
MS Level for identification	MS1, MS2	Check on:	Isomeric overlap, Isobaric overlap, In-source fragmentation
Identification level	Molecular species level	Limit of detection	Signal theshold
Polarity mode	Negative	RT verified by standard	Yes
Type of negative (precursor)ion	[M-H]-	Separation of isobaric/isomeric interferece confirmed	No
Fragments for identification		Model for separation prediction	No

Fragment name
FA(-H)
HG(PI,241)
GP(153)
-(C6H12O6, 180)

Isotope correction at MS1	No	Additional dimension/techniques	-
Isotope correction at MS2	No	Lipid Identification Software	LipidHunter
MS1 verified by standard	No	Data manipulation	-
MS2 verified by standard	No	Nomenclature for intact lipid molecule	Yes
Background check at MS1	Yes	Nomenclature for fragment ions	N/A
Background check at MS2	Yes	Further identification remarks	Proposed identifications were further validated by plotting the retention time of lipid species against their Kendrick mass defect by hydrogen and lipid species not following expected trendlines were excluded.

7) LPI[M-H]- / Lipid quantification

Quantitative	Yes	Type I isotope correction	Yes
MS Level for quantification	MS1, MS2	Limit of quantification	Linear regression analysis was applied by plotting the calculated concentrations against their AUC value to identify and exclude possible outliers and features showing non-linear behaviour.
Internal lipid standard(s) MS1		Normalization to reference	Yes

Internal standard	Endogenous subclass
LPE 18:1 (d7)	LPI

Internal lipid standard(s) MS2		Lipid Quantification Software	Skyline

Internal standard	Fragment(s)	Endogenous subclass
LPE 18:1 (d7)	FA(-H)	LPI
LPE 18:1 (d7)	HG(PI,241)	LPI
LPE 18:1 (d7)	GP(153)	LPI
LPE 18:1 (d7)	-(C6H12O6, 180)	LPI

Type of quantification	Internal standard amount	Batch correction	No
Response correction	No	Further quantification remarks	Individual lipid species concentrations were normalized to the wet tissue weight.

8) LPS[M-H]- / Lipid identification

Lipid class	LPS	Did you presume assumptions for identification?	No
MS Level for identification	MS1, MS2	Check on:	Isomeric overlap, Isobaric overlap, In-source fragmentation
Identification level	Molecular species level	Limit of detection	Signal theshold
Polarity mode	Negative	RT verified by standard	Yes
Type of negative (precursor)ion	[M-H]-	Separation of isobaric/isomeric interferece confirmed	No
Fragments for identification		Model for separation prediction	No
Isotope correction at MS1	No	Additional dimension/techniques	-
Isotope correction at MS2	No	Lipid Identification Software	LipidHunter
MS1 verified by standard	No	Data manipulation	-
MS2 verified by standard	No	Nomenclature for intact lipid molecule	Yes
Background check at MS1	Yes	Nomenclature for fragment ions	N/A
Background check at MS2	Yes	Further identification remarks	Proposed identifications were further validated by plotting the retention time of lipid species against their Kendrick mass defect by hydrogen and lipid species not following expected trendlines were excluded.

Fragments for identification:

Fragment name
FA1(-H)
-(C3H5NO2,87)
GP(153)

8) LPS[M-H]- / Lipid quantification

Quantitative	Yes	Type I isotope correction	Yes
MS Level for quantification	MS1, MS2	Limit of quantification	Linear regression analysis was applied by plotting the calculated concentrations against their AUC value to identify and exclude possible outliers and features showing non-linear behaviour.
Internal lipid standard(s) MS1		Normalization to reference	Yes

Internal standard	Endogenous subclass
LPE 18:1 (d7)	LPS

Internal lipid standard(s) MS2		Lipid Quantification Software	Skyline

Internal standard	Fragment(s)	Endogenous subclass
LPE 18:1 (d7)	FA1(-H)	LPS
LPE 18:1 (d7)	-(C3H5NO2,87)	LPS
LPE 18:1 (d7)	GP(153)	LPS

Type of quantification	Internal standard amount	Batch correction	No
Response correction	No	Further quantification remarks	Individual lipid species concentrations were normalized to the wet tissue weight.

9) GM3[M-H]- / Lipid identification

Lipid class	GM3	Did you presume assumptions for identification?	No
MS Level for identification	MS1, MS2	Check on:	Isomeric overlap, Isobaric overlap, In-source fragmentation
Identification level	Species level	Limit of detection	Signal theshold
Polarity mode	Negative	RT verified by standard	Yes
Type of negative (precursor)ion	[M-H]-	Separation of isobaric/isomeric interferece confirmed	No
Fragments for identification		Model for separation prediction	No

Fragment name
-HG(NHex,291)
HG(NHex,290)
-HG(NHex3,615)

Isotope correction at MS1	No	Additional dimension/techniques	-
Isotope correction at MS2	Type 2	Lipid Identification Software	manually
MS1 verified by standard	Yes	Data manipulation	-
MS2 verified by standard	Yes	Nomenclature for intact lipid molecule	Yes
Background check at MS1	Yes	Nomenclature for fragment ions	N/A
Background check at MS2	Yes	Further identification remarks	Proposed identifications were further validated by plotting the retention time of lipid species against their Kendrick mass defect by hydrogen and lipid species not following expected trendlines were excluded.

9) GM3[M-H]- / Lipid quantification

Quantitative	Yes	Limit of quantification	Linear regression analysis was applied by plotting the calculated concentrations against their AUC value to identify and exclude possible outliers and features showing non-linear behaviour.
MS Level for quantification	MS2	Normalization to reference	Yes
Internal lipid standard(s) MS2		Lipid Quantification Software	Skyline

Internal standard	Fragment(s)	Endogenous subclass
GlcCer d18:1;O2/12:0	-HG(NHex,291)	GM3
GlcCer d18:1;O2/12:0	HG(NHex,290)	GM3
GlcCer d18:1;O2/12:0	-HG(NHex3,615)	GM3

Type of quantification	Internal standard amount	Batch correction	No
Response correction	No	Further quantification remarks	Individual lipid species concentrations were normalized to the wet tissue weight.
Type I isotope correction	Yes		

Supplementary Note 3 | Lipidomics Minimal Reporting Checklist with details on the AV lipid quantification using acidified methanol/chloroform extraction method and targeted lipidomics workflow.

Created by https://lipidomicstandards.org, version v2.4.0

Contents of Report

Separation Workflow

Overall study design

Title of the study	Sex-specific lipidomic signatures in aortic valve disease reflect differential fibro-calcific progression (targeted, acidified MeOH/CHCl3)		
Document creation date	12/20/2024	Corresponding Email	maria.fedorova@tu-dresden.de
Principal investigator	Maria Fedorova	Is the workflow targeted or untargeted?	Targeted
Institution	Technical University Dresden, Germany	Clinical	No

Lipid extraction

Extraction method	2-phase system	Were internal standards added prior extraction?	Yes
pH adjustment	Hydrochloric acid	Special conditions	All solvents used for extraction were supplemented with 0.1 % (w/v) BHT. All extraction steps were performed on ice.
2-phase system	Folch	Derivatization	-

Analytical platform

Ionizaton additives	Ammonium formate, Formic acid	Mass resolution for detected ion at MS1	Low resolution
Number of separation dimensions	One dimension	Resolution at MS1	Unit
Separation type 1	LC	Recording mode of raw data at MS1	Profile mode
Separation mode 1 (liquid)	RP	Mass window for precursor ion isolation (in Da total isolation window)	0.7
Detector	Mass spectrometer	Mass resolution for detected ion at MS2	Low resolution
MS type	QQQ	Resolution at MS2	Unit
MS vendor	Thermo	Recording mode of raw data at MS2	Profile mode
Ion source	ESI	Was/Were additional dimension/techniques used	No
MS Level	MS1, MS2		

Quality control

Blanks	Yes	Quality control	Yes
Type of Blanks	Solvent blank, Internal standard blank	Type of QC sample	Sample pool

Method qualification and validation

Method validation	Yes	Precision	No
Lipid recovery	Yes	Accuracy	No
Dynamic quantification range	Yes	Guidelines followed	None
Limit of quantitation (LOQ)/Limit of detection (LOD)	Yes		

Reporting

Are reported raw data uploaded into repository?	Yes	Summary data	Quantification and identification data
Link to repository / ID to entry	doi: 10.21228/M8G24F	Raw data upload	Yes
Are metadata available?	Yes	Additional comments	-

Sample Descriptions

Aortic valve tissue / Human / Tissues (e.g., liver, heart, brain)

Perfusion	No	Additives	None
Storage and collection conditions	Available	Were samples stored under inert gas?	No
Provided preanalytical information	Freeze-thaw cycles	Additional preservation methods	No
Temperature handling original sample	Room temperature	Biobank samples	No
Instant sample preparation	No	Sample homogenization	Yes
Storage temperature	-80 °C	Sample homogenization solvent	liquid nitrogen
Freeze-thaw cycles	1		

Lipid Class Descriptions

1) PA[M-H]- / Lipid identification

Lipid class	PA	Which assumptions were presumed?	It was checked that the identified PA is of endogeneous origin and not the result of in-source fragmentation of other PL.
MS Level for identification	MS1, MS2	Check on:	Isomeric overlap, Isobaric overlap, In-source fragmentation
Identification level	Molecular species level	Limit of detection	Signal theshold
Polarity mode	Negative	RT verified by standard	Yes
Type of negative (precursor)ion	[M-H]-	Separation of isobaric/isomeric interferece confirmed	No
Fragments for identification		Model for separation prediction	No

Fragment name
FA1(-H)
FA2(-H)
GP(153)
P(79)

Isotope correction at MS1	No	Additional dimension/techniques	-
Isotope correction at MS2	No	Lipid Identification Software	manually
MS1 verified by standard	Yes	Data manipulation	-
MS2 verified by standard	Yes	Nomenclature for intact lipid molecule	Yes
Background check at MS1	Yes	Nomenclature for fragment ions	N/A
Background check at MS2	Yes	Further identification remarks	Proposed identifications were further validated by plotting the retention time of lipid species against their Kendrick mass defect by hydrogen and lipid species not following expected trendlines were excluded.
Did you presume assumptions for identification?	Yes		

1) PA[M-H]- / Lipid quantification

Quantitative	Yes	Limit of quantification	Linear regression analysis was applied by plotting the calculated concentrations against their AUC value to identify and exclude possible outliers and features showing non-linear behaviour.
MS Level for quantification	MS2	Normalization to reference	Yes
Internal lipid standard(s) MS2		Lipid Quantification Software	Skyline

Internal standard	Fragment(s)	Endogenous subclass
PA 15:0/18:1 (d7)	FA1(-H)	PA
PA 15:0/18:1 (d7)	FA2(-H)	PA
PA 15:0/18:1 (d7)	GP(153)	PA

Type of quantification	Internal standard amount	Batch correction	No
Response correction	No	Further quantification remarks	Individual lipid species concentrations were normalized to the wet tissue weight.
Type I isotope correction	Yes		

2) PS[M-H]- / Lipid identification

Lipid class	PS	Did you presume assumptions for identification?	No
MS Level for identification	MS1, MS2	Check on:	Isomeric overlap, Isobaric overlap, In-source fragmentation
Identification level	Molecular species level	Limit of detection	Signal theshold
Polarity mode	Negative	RT verified by standard	Yes
Type of negative (precursor)ion	[M-H]-	Separation of isobaric/isomeric interferece confirmed	No
Fragments for identification		Model for separation prediction	No

Fragment name
-(C3H5NO2,87)
FA1(-H)
FA2(-H)
GP(153)

Isotope correction at MS1	No	Additional dimension/techniques	-
Isotope correction at MS2	Type 2	Lipid Identification Software	LipidHunter
MS1 verified by standard	Yes	Data manipulation	-
MS2 verified by standard	Yes	Nomenclature for intact lipid molecule	Yes
Background check at MS1	Yes	Nomenclature for fragment ions	N/A
Background check at MS2	Yes	Further identification remarks	Proposed identifications were further validated by plotting the retention time of lipid species against their Kendrick mass defect by hydrogen and lipid species not following expected trendlines were excluded.

2) PS[M-H]- / Lipid quantification

Quantitative	Yes	Limit of quantification	Linear regression analysis was applied by plotting the calculated concentrations against their AUC value to identify and exclude possible outliers and features showing non-linear behaviour.
MS Level for quantification	MS2	Normalization to reference	Yes
Internal lipid standard(s) MS2		Lipid Quantification Software	Skyline
Type of quantification	Internal standard amount	Batch correction	No
Response correction	No	Further quantification remarks	Individual lipid species concentrations were normalized to the wet tissue weight.
Type I isotope correction	Yes		

Internal standard	Fragment(s)	Endogenous subclass
PS 15:0/18:1 (d7)	-(C3H5NO2,87)	PS
PS 15:0/18:1 (d7)	FA1(-H)	PS
PS 15:0/18:1 (d7)	FA2(-H)	PS
PS 15:0/18:1 (d7)	GP(153)	PS

3) PI[M-H]- / Lipid identification

Lipid class	PI	Did you presume assumptions for identification?	No
MS Level for identification	MS1, MS2	Check on:	Isomeric overlap, Isobaric overlap, In-source fragmentation
Identification level	Molecular species level	Limit of detection	Signal theshold
Polarity mode	Negative	RT verified by standard	Yes
Type of negative (precursor)ion	[M-H]-	Separation of isobaric/isomeric interferece confirmed	No
Fragments for identification	Fragment name: FA1(-H); FA2(-H); HG(PI,241)-(H2O); -FA1(-H); -FA2(-H)	Model for separation prediction	No
Isotope correction at MS1	No	Additional dimension/techniques	-
Isotope correction at MS2	Type 2	Lipid Identification Software	LipidHunter
MS1 verified by standard	Yes	Data manipulation	-
MS2 verified by standard	Yes	Nomenclature for intact lipid molecule	Yes
Background check at MS1	Yes	Nomenclature for fragment ions	N/A
Background check at MS2	Yes	Further identification remarks	Proposed identifications were further validated by plotting the retention time of lipid species against their Kendrick mass defect by hydrogen and lipid species not following expected trendlines were excluded.

3) PI[M-H]- / Lipid quantification

Quantitative	Yes	Limit of quantification	Linear regression analysis was applied by plotting the calculated concentrations against their AUC value to identify and exclude possible outliers and features showing non-linear behaviour.
MS Level for quantification	MS2	Normalization to reference	Yes
Internal lipid standard(s) MS2		Lipid Quantification Software	Skyline

Internal standard	Fragment(s)	Endogenous subclass
PI 15:0/18:1 (d7)	FA1(-H)	PI
PI 15:0/18:1 (d7)	FA2(-H)	PI
PI 15:0/18:1 (d7)	HG(PI,241)-(H2O)	

Type of quantification	Internal standard amount	Batch correction	No
Response correction	No	Further quantification remarks	Individual lipid species concentrations were normalized to the wet tissue weight.
Type I isotope correction	Yes		

4) PG[M-H]- / Lipid identification

Lipid class	PG	Did you presume assumptions for identification?	No
MS Level for identification	MS1, MS2	Check on:	Isomeric overlap, Isobaric overlap, In-source fragmentation
Identification level	Molecular species level	Limit of detection	Signal theshold
Polarity mode	Negative	RT verified by standard	Yes
Type of negative (precursor)ion	[M-H]-	Separation of isobaric/isomeric interferece confirmed	No
Fragments for identification		Model for separation prediction	No

Fragment name
FA1(-H)
FA2(-H)
GP(153)
-FA1(+HO)
-FA2(+HO)

Isotope correction at MS1	No	Additional dimension/techniques	-
Isotope correction at MS2	Type 2	Lipid Identification Software	LipidHunter
MS1 verified by standard	Yes	Data manipulation	-
MS2 verified by standard	Yes	Nomenclature for intact lipid molecule	Yes
Background check at MS1	Yes	Nomenclature for fragment ions	N/A
Background check at MS2	Yes	Further identification remarks	Proposed identifications were further validated by plotting the retention time of lipid species against their Kendrick mass defect by hydrogen and lipid species not following expected trendlines were excluded.

4) PG[M-H]- / Lipid quantification

Quantitative	Yes	Limit of quantification	Linear regression analysis was applied by plotting the calculated concentrations against their AUC value to identify and exclude possible outliers and features showing non-linear behaviour.
MS Level for quantification	MS2	Normalization to reference	Yes
Internal lipid standard(s) MS2		Lipid Quantification Software	Skyline

Internal standard	Fragment(s)	Endogenous subclass
PG 15:0/18:1 (d7)	FA1(-H)	PG
PG 15:0/18:1 (d7)	FA2(-H)	PG
PG 15:0/18:1 (d7)	GP(153)	PG

Type of quantification	Internal standard amount	Batch correction	No
Response correction	No	Further quantification remarks	Individual lipid species concentrations were normalized to the wet tissue weight.
Type I isotope correction	Yes		

5) CL[M-H]- / Lipid identification

Lipid class	CL	Did you presume assumptions for identification?	No
MS Level for identification	MS1, MS2	Check on:	Isomeric overlap, Isobaric overlap, In-source fragmentation
Identification level	Molecular species level	Limit of detection	Signal theshold
Polarity mode	Negative	RT verified by standard	Yes
Type of negative (precursor)ion	[M-H]-	Separation of isobaric/isomeric interferece confirmed	No
Fragments for identification		Model for separation prediction	No

Fragment name
-PG
-PG-FA1
-PG-FA1-(H2O)
FA1(-H)
FA2(-H)
GP(153)

Isotope correction at MS1	No	Additional dimension/techniques	-
Isotope correction at MS2	Type 2	Lipid Identification Software	manually
MS1 verified by standard	Yes	Data manipulation	-
MS2 verified by standard	Yes	Nomenclature for intact lipid molecule	Yes
Background check at MS1	Yes	Nomenclature for fragment ions	N/A
Background check at MS2	Yes	Further identification remarks	Proposed identifications were further validated by plotting the retention time of lipid species against their Kendrick mass defect by hydrogen and lipid species not following expected trendlines were excluded.

5) CL[M-H]- / Lipid quantification

Quantitative	Yes	Limit of quantification	Linear regression analysis was applied by plotting the calculated concentrations against their AUC value to identify and exclude possible outliers and features showing non-linear behaviour.
MS Level for quantification	MS2	Normalization to reference	Yes
Internal lipid standard(s) MS2		Lipid Quantification Software	Skyline
Type of quantification	Internal standard amount	Batch correction	No
Response correction	No	Further quantification remarks	Individual lipid species concentrations were normalized to the wet tissue weight.
Type I isotope correction	Yes		

Internal lipid standard(s) MS2:

Internal standard	Fragment(s)	Endogenous subclass
CL 18:2/18:2/18:2/18:2 (d5)	FA1(-H)	CL
CL 18:2/18:2/18:2/18:2 (d5)	FA2(-H)	CL
CL 18:2/18:2/18:2/18:2 (d5)	GP(153)	CL

6) LPA[M-H]- / Lipid identification

Lipid class	LPA	Did you presume assumptions for identification?	No
MS Level for identification	MS1, MS2	Check on:	Isomeric overlap, Isobaric overlap, In-source fragmentation
Identification level	Molecular species level	Limit of detection	Signal theshold
Polarity mode	Negative	RT verified by standard	Yes
Type of negative (precursor)ion	[M-H]-	Separation of isobaric/isomeric interferece confirmed	No
Fragments for identification		Model for separation prediction	No
Isotope correction at MS1	No	Additional dimension/techniques	-
Isotope correction at MS2	No	Lipid Identification Software	manually
MS1 verified by standard	Yes	Data manipulation	-
MS2 verified by standard	Yes	Nomenclature for intact lipid molecule	Yes
Background check at MS1	Yes	Nomenclature for fragment ions	N/A
Background check at MS2	Yes	Further identification remarks	Proposed identifications were further validated by plotting the retention time of lipid species against their Kendrick mass defect by hydrogen and lipid species not following expected trendlines were excluded.

Fragments for identification:

Fragment name
FA1(-H)
GP(153)
P(79)

6) LPA[M-H]- / Lipid quantification

Quantitative	Yes	Limit of quantification	Linear regression analysis was applied by plotting the calculated concentrations against their AUC value to identify and exclude possible outliers and features showing non-linear behaviour.
MS Level for quantification	MS2	Normalization to reference	Yes
Internal lipid standard(s) MS2		Lipid Quantification Software	Skyline
Type of quantification	Internal standard amount	Batch correction	No
Response correction	No	Further quantification remarks	Individual lipid species concentrations were normalized to the wet tissue weight.
Type I isotope correction	Yes		

Internal lipid standard(s) MS2:

Internal standard	Fragment(s)	Endogenous subclass
LPA 17:1	FA1(-H)	LPA
LPA 17:1	GP(153)	LPA
LPA 17:1	P(79)	LPA

7) LPI[M-H]- / Lipid identification

Lipid class	LPI	Did you presume assumptions for identification?	No
MS Level for identification	MS1, MS2	Check on:	Isomeric overlap, Isobaric overlap, In-source fragmentation
Identification level	Molecular species level	Limit of detection	Signal theshold
Polarity mode	Negative	RT verified by standard	Yes
Type of negative (precursor)ion	[M-H]-	Separation of isobaric/isomeric interferece confirmed	No
Fragments for identification		Model for separation prediction	No

Fragment name
FA(-H)
HG(PI,241)
GP(153)
-(C6H12O6, 180)

Isotope correction at MS1	No	Additional dimension/techniques	-
Isotope correction at MS2	No	Lipid Identification Software	LipidHunter
MS1 verified by standard	No	Data manipulation	-
MS2 verified by standard	No	Nomenclature for intact lipid molecule	Yes
Background check at MS1	Yes	Nomenclature for fragment ions	N/A
Background check at MS2	Yes	Further identification remarks	Proposed identifications were further validated by plotting the retention time of lipid species against their Kendrick mass defect by hydrogen and lipid species not following expected trendlines were excluded.

7) LPI[M-H]- / Lipid quantification

Quantitative	Yes	Type I isotope correction	Yes
MS Level for quantification	MS1, MS2	Limit of quantification	Linear regression analysis was applied by plotting the calculated concentrations against their AUC value to identify and exclude possible outliers and features showing non-linear behaviour.
Internal lipid standard(s) MS1		Normalization to reference	Yes

Internal standard	Endogenous subclass
LPE 18:1 (d7)	LPI

Internal lipid standard(s) MS2		Lipid Quantification Software	Skyline

Internal standard	Fragment(s)	Endogenous subclass
LPE 18:1 (d7)	FA(-H)	LPI
LPE 18:1 (d7)	HG(PI,241)	LPI
LPE 18:1 (d7)	GP(153)	LPI
LPE 18:1 (d7)	-(C6H12O6, 180)	LPI

Type of quantification	Internal standard amount	Batch correction	No
Response correction	No	Further quantification remarks	Individual lipid species concentrations were normalized to the wet tissue weight.

8) LPS[M-H]- / Lipid identification

Lipid class	LPS	Did you presume assumptions for identification?	No
MS Level for identification	MS1, MS2	Check on:	Isomeric overlap, Isobaric overlap, In-source fragmentation
Identification level	Molecular species level	Limit of detection	Signal theshold
Polarity mode	Negative	RT verified by standard	Yes
Type of negative (precursor)ion	[M-H]-	Separation of isobaric/isomeric interferece confirmed	No
Fragments for identification	Fragment name: FA1(-H); -(C3H5NO2,87); GP(153)	Model for separation prediction	No
Isotope correction at MS1	No	Additional dimension/techniques	-
Isotope correction at MS2	No	Lipid Identification Software	LipidHunter
MS1 verified by standard	No	Data manipulation	-
MS2 verified by standard	No	Nomenclature for intact lipid molecule	Yes
Background check at MS1	Yes	Nomenclature for fragment ions	N/A
Background check at MS2	Yes	Further identification remarks	Proposed identifications were further validated by plotting the retention time of lipid species against their Kendrick mass defect by hydrogen and lipid species not following expected trendlines were excluded.

8) LPS[M-H]- / Lipid quantification

Quantitative	Yes	Type I isotope correction	Yes
MS Level for quantification	MS1, MS2	Limit of quantification	Linear regression analysis was applied by plotting the calculated concentrations against their AUC value to identify and exclude possible outliers and features showing non-linear behaviour.
Internal lipid standard(s) MS1		Normalization to reference	Yes

Internal standard	Endogenous subclass
LPE 18:1 (d7)	LPS

Internal lipid standard(s) MS2		Lipid Quantification Software	Skyline

Internal standard	Fragment(s)	Endogenous subclass
LPE 18:1 (d7)	FA1(-H)	LPS
LPE 18:1 (d7)	-(C3H5NO2,87)	LPS
LPE 18:1 (d7)	GP(153)	LPS

Type of quantification	Internal standard amount	Batch correction	No
Response correction	No	Further quantification remarks	Individual lipid species concentrations were normalized to the wet tissue weight.

9) GM3[M-H]- / Lipid identification

Lipid class	GM3	Did you presume assumptions for identification?	No
MS Level for identification	MS1, MS2	Check on:	Isomeric overlap, Isobaric overlap, In-source fragmentation
Identification level	Species level	Limit of detection	Signal theshold
Polarity mode	Negative	RT verified by standard	Yes
Type of negative (precursor)ion	[M-H]-	Separation of isobaric/isomeric interferece confirmed	No
Fragments for identification	Fragment name: -HG(NHex,291); HG(NHex,290); -HG(NHex3,615)	Model for separation prediction	No
Isotope correction at MS1	No	Additional dimension/techniques	-
Isotope correction at MS2	Type 2	Lipid Identification Software	manually
MS1 verified by standard	Yes	Data manipulation	-
MS2 verified by standard	Yes	Nomenclature for intact lipid molecule	Yes
Background check at MS1	Yes	Nomenclature for fragment ions	N/A
Background check at MS2	Yes	Further identification remarks	Proposed identifications were further validated by plotting the retention time of lipid species against their Kendrick mass defect by hydrogen and lipid species not following expected trendlines were excluded.

9) GM3[M-H]- / Lipid quantification

Quantitative	Yes	Limit of quantification	Linear regression analysis was applied by plotting the calculated concentrations against their AUC value to identify and exclude possible outliers and features showing non-linear behaviour.
MS Level for quantification	MS2	Normalization to reference	Yes
Internal lipid standard(s) MS2		Lipid Quantification Software	Skyline
Type of quantification	Internal standard amount	Batch correction	No
Response correction	No	Further quantification remarks	Individual lipid species concentrations were normalized to the wet tissue weight.
Type I isotope correction	Yes		

Internal lipid standard(s) MS2:

Internal standard	Fragment(s)	Endogenous subclass
GlcCer d18:1;O2/12:0	-HG(NHex,291)	GM3
GlcCer d18:1;O2/12:0	HG(NHex,290)	GM3
GlcCer d18:1;O2/12:0	-HG(NHex3,615)	GM3
