# Supplementary material for: Sex-specific lipidomic signatures in aortic valve disease reflect differential fibro-calcific progression
Source: Nat Commun. 2025 Jun 3;16:5163. doi: 10.1038/s41467-025-60411-2 (PMC12134101; doi:10.1038/s41467-025-60411-2)
Supplement: Supplementary file 2 — Description of Additional Supplementary Files [file 41467_2025_60411_MOESM2_ESM.docx]

**Supplementary Data**

**Supplementary Data 1 | Clinical metadata. a,** Number of samples per group. Median [IQR] or total count per group (%). **b,** Baseline parameters per sex in tricuspid aortic valves. Median [IQR] or total count per group (%). P values were calculated by two- sided T-test. **c,** Baseline parameters per sex in all patients. Median [IQR] or total count

per group (%). P values were calculated by two-sided T-test. **d,** Baseline parameters per valve morphology. Median [IQR] or total count per group (%). P values were calculated by two-sided T-test.

**Supplementary Data 2 | Lipid molecular species identified by software assisted and/or manual annotation.**

**Supplementary Data 3 | Quantitative values of individual lipids in human aortic valve tissue extracts [pmol/mg wet tissue weight].**

**Supplementary Data 4 | One-way ANOVA & post-hoc results comparing TAV and BAV lipidomes along different stages of FCAVD development. a,** statistical comparison of mildly diseases, fibrotic and calcified lipidomes of TAV; **b,** statistical comparison of male and female mildly diseases, fibrotic and calcified lipidomes of TAV; **c,** statistical comparison of male and female mildly diseases, fibrotic and calcified lipidomes of TAV and BAV.

**Supplementary Data 5 | Pairwise comparison of significantly regulated lipids** (determined by Fold Change (FC) Analysis > 1.5 and two-sided T-test P < 0.05) between (**a**) female versus male sex in TAV tissue at mildly diseased, fibrotic stage and calcific stage as well as between (**b**) TAV versus BAV morphology at mildly diseased, fibrotic and calcific stage.

**Supplementary Data 6 | Internal standard mixtures (ISTD) used for qHPTLC lipid class specific quantification.** Calibration curves were built to roughly estimate the composition and relative abundance of aortic valve (AV) lipids in order to design ISTD mixture tailored to the lipid subclasses and their endogenous quantities in human AV lipidome.

**Supplementary Data 7 | Serial dilutions of AV-tailored ISTD mixture used for seven-point calibration curves generation.** Provided ISTD quantities correspond to 10 μL of the mixture spiked in 20 mg of AV tissue. Selected amounts are marked in bold.

**Supplementary Data 8 | Composition of the AV-tailored ISTD mixture.** Provided ISTD quantities correspond to 10 μL of the mixture spiked in 20 mg of AV tissue.

**Supplementary Data 9 | Adducts and common in-source fragments considered for peak integration to support accurate lipid quantification.** * [M+Na]+ Adduct of Hex1Cer lipid species was not considered, due to an overlap of the corresponding m/z values with an unidentified species.

**Supplementary Data 10 | Lipid-specific selected reaction monitoring (SRM) transitions.**

**Supplementary Data 11 | Uniform Manifold Approximation and Projection (UMAP) parameters.**
